# Cross‐Disease Classification of Depression, Bipolar Disorder, and Schizophrenia Using Multiscale Spatiotemporal Network Fusion Features From fNIRS

**DOI:** 10.1155/da/9921579

**Published:** 2026-10-06

**Authors:** Yajing Meng, Yulu Yang, Qing Li, Qian Fu, Mingyao Gao, Ling He, Jing Zhang, Changjian Qiu

**Affiliations:** ^1^ Mental Health Center West China Hospital of Sichuan University Chengdu 610041 China wchscu.cn; ^2^ College of Biomedical Engineering Sichuan University Chengdu 610065 China scu.edu.cn

**Keywords:** functional near–infrared spectroscopy, machine learning, neuroimaging biomarkers, verbal fluency task

## Abstract

**Background:**

Major depressive disorder (MDD), bipolar disorder (BD), and schizophrenia (SCZ) are highly prevalent and disabling psychiatric conditions with increasing incidence, significantly affecting individuals’ daily functioning, social relationships, and overall quality of life. Current diagnostic practices largely rely on clinical interviews and symptom‐based rating scales, which are inherently subjective and time‐consuming—posing major obstacles to large‐scale screening.

**Methods:**

To address this, we propose a novel multiscale spatiotemporal network fusion (MSNF) feature extraction method based on functional near–infrared spectroscopy (fNIRS) combined with the verbal fluency test (VFT). This method is designed to enable objective, efficient, and automated differentiation among multiple psychiatric disorders (MDD, BD, and SCZ), as well as between patient groups and healthy controls (HCs). Leveraging the practical advantages of fNIRS, including its portability, motion tolerance, and balanced spatiotemporal resolution, the proposed MSNF fuses temporal dynamics with graph‐theoretical topology, capturing both local hemodynamic dynamics and hierarchy connectivity patterns.

**Results:**

In this study, a total of 414 participants were recruited, including individuals diagnosed with MDD, BD, SCZ, and HCs. Classification experiments using machine learning (ML) models demonstrate the proposed method’s robustness and superior diagnostic performance in binary and multiclass settings, outperforming traditional unidimensional feature approaches. Additionally, group‐level functional connectivity (FC) analyses reveal distinct neurobiological signatures among psychiatric groups.

**Conclusions:**

These findings underscore the translational potential of MSNF‐enhanced fNIRS as a scalable neuroimaging–based biomarker tool for early psychiatric screening and differential diagnosis.

## 1. Introduction

Major depressive disorder (MDD), bipolar disorder (BD), and schizophrenia (SCZ) are highly prevalent and disabling psychiatric disorders, with their prevalence demonstrating a sustained upward trajectory in recent years [1–5]. These disorders impose heavy societal burden [6, 7], severely limit psychosocial functioning, and reduce quality of life [8], manifesting through multifaceted consequences including social isolation, stigma, and other negative outcomes [9].

Currently, the clinical diagnosis of mental disorders primarily relies on psychological scales and clinicians’ observations of patients’ symptoms, behavioral patterns, and functional changes, following the diagnostic criteria outlined in the Diagnostic and Statistical Manual of Mental Disorders (5th edition, DSM‐5) [10–12]. However, this approach exhibits significant subjectivity and is susceptible to multiple limitations, including the heterogeneity of individual functional and behavioral manifestations and variations in clinicians’ professional expertise [13]. These limitations collectively underscore the urgent need to develop objective biomarkers to enhance diagnostic precision in psychiatric practice.

Alterations in brain networks have been implicated in MDD, BD, and SCZ [14]. Abnormal functional connectivity (FC) is increasingly recognized as a promising candidate marker for the clinical diagnosis of severe mental disorders [15, 16]. Psychiatric patients have diminished FC strength in specific brain regions, disrupted connectivity within frontal‐parietal networks, and a pattern of disorganized neural circuits in regions and networks that play a key role in adaptive emotional responses and regulation [16, 17]. Comparative analyses revealed disease‐specific FC characteristics, with a shift in the network structure of patients towards randomized configurations, with SCZ patients having the highest degree of randomization, followed by BD patients, and MDD patients having the lowest degree of randomization, as well as a significant decrease in short‐range connectivity and a significant increase in medium‐range/long‐range connectivity among the three categories of patients [14]. Connectivity in the prefrontal cortex was significantly reduced in people with SCZ compared to depressed patients and healthy controls (HCs) [18]. The characterization of common and differential connectivity in functional networks expands the understanding of common and differential pathophysiological mechanisms and provides important insights into the development of psychiatric disease–specific biomarkers.

Neuroimaging provides an important research paradigm for resolving neuropathologic differences in psychiatric disorders. Structural neuroimaging techniques such as magnetic resonance imaging (MRI) [19] and functional neuroimaging techniques such as functional MRI (fMRI) [20], electroencephalography (EEG) [21], and functional near–infrared spectroscopy (fNIRS) [10, 22] are able to distinguish patients from HCs as an adjunct to diagnosing these disorders.

By detecting changes in the concentration of oxygenated/deoxygenated hemoglobin (HbO/HbR), fNIRS combines the advantages of ease of wear, environmental tolerance, and eco‐efficacy while ensuring high temporal resolution, with enhanced tolerance to motion artifacts [23–25]. Its spatial resolution is significantly better than that of EEG, and its temporal resolution is better than that of fMRI [26]. fNIRS most often constitutes a standardized paradigm with the verbal fluency test (VFT), which elicits different abnormalities associated with each of the major diagnostic groups of mental disorders [22, 27]. Based on this, Liu et al. [26] used the VFT to study the differences between depressed adolescents and HCs in brain differences in activity patterns and found that the depressed adolescent group presented abnormal prefrontal activation patterns and attenuation of FC strength when performing this task, providing neurological evidence for the study of cognitive neurological functioning in depressed adolescents. Wang et al. [22] demonstrated that the VFT paradigm holds promise for detecting and differentiating psychiatric disorders and has the potential to develop accessible neuroimaging biomarkers for different psychiatric disorders.

Machine learning (ML) is able to learn from data and achieve prediction and recognition of unknown data through automatic iteration for classification tasks [28]. Numerous studies have demonstrated the ability to mine potential features of structural or functional changes in the brain using ML methods with the help of fNIRS techniques [29], thus enabling cross‐disease diagnosis [30]. Wei et al. [31] constructed a binary logistic regression model by monitoring the dynamic changes of HbO in the bilateral temporo‐frontal coalition area in the test of Chinese verbal fluency and used the integral value and the center of mass value as the biometric feature, and the area under the curve (AUC) of the working characteristics of the subjects in the psychiatric illness group and the HC group amounted to 0.913, of which the discriminant AUC of the subgroup of the mood disorders was 0.899 and 0.737 for the SCZ subgroup. Diao et al. [32] used the fNIRS technique to measure brain activation after fNIRS of the VFT task state and constructed a SCZ‐MDD‐HC triple categorization by combining five ML algorithms with 77 dimensional time–frequency feature extraction, achieving an overall accuracy of 85.90% (AUC = 0.95). Eken et al. [33] used the Reading the Mind from the Eyes Test to monitor hemodynamics in the frontal cortex by fNIRS and applied support vector machine (SVM), discriminant analysis (DA), and K‐nearest neighbor (KNN) algorithms to differentially classify the SCZ from the BD, ultimately obtaining a cross‐diagnostic accuracy of 75.5%. In the current ML‐based fNIRS research, there are dimensional limitations and insufficient depth of characterization in the construction of the feature space. Most of the studies are limited to shallow statistics (e.g., mean and variance) of a single time series or traditional frequency domain indexes, which can measure the overall characteristics of the data but fail to fully explore the multiscale characteristics of the signals, which results in the challenges of extracting the deep information of the physiological signals [34].

## 2. Materials and Methods

This study presented an fNIRS‐based classification pipeline for identifying three major psychiatric disorders using data collected during a VFT. After preprocessing the fNIRS signals, we extracted temporal local hemoglobin dynamics and constructed functional brain networks to derive spatial hierarchy connectivity pattern. These multiscale features were fused and used for classification via ML models, including random forest (RF), SVM, and multilayer perceptron (MLP). Additionally, brain network analysis was conducted to explore connectivity alterations across groups, providing both diagnostic performance and neurobiological insight.

### 2.1. Participants

We recruited 414 participants for the study. The participant recruitment, screening, exclusion, and inclusion process are summarized in Figure S1. All subjects satisfied the criteria of the HC group, the depression group (MDD), the SCZ group, and the BD group. Detailed demographic and clinical characteristics, including illness duration, age of onset, and medication status, are summarized in Table 1. The patients in our study were all diagnosed using DSM‐5 by three senior psychiatrists. Ultimately, the participant distribution comprised 103 BD individuals, 105 MDD individuals, 102 SCZ individuals, and 104 HCs. Our samples were sourced from outpatient and inpatient departments. For inpatients, we used various observer‐rating scales because they were in an acute phase of illness, including Hamilton Depression Rating Scale (HAMD), Hamilton Anxiety Rating Scale (HAMA), Young Mania Rating Scale (YMRS), and Positive and Negative Syndrome Scale (PANSS) [35]. For outpatients, we used self‐report scales as most were in a stable phase, assuming they could complete the questionnaires, including Self‐rating Depression Scale (SDS), Self‐rating Anxiety Scale (SAS), and Hypomania Checklist‐32 (HCL‐32). However, upon collecting the questionnaires, we found that, due to various reasons, these patients filled them out improperly or even refused to complete the self‐report scales. Among the patients with BD, there were four different states: manic episode (*n* = 14), depressive episode (*n* = 12), mixed state (*n* = 21), and stable state (*n* = 56). We chose not to consider the dimension of whether patients had psychotic symptoms. Although patients with MDD were clinically diagnosed with unipolar depressive episodes, some of their mania scale scores reached the threshold levels (*n* = 13, the optimal YMRS severity threshold of 25 was determined) [36], so BD cannot be ruled out. This is especially true for adolescent and early adult patients, as Chinese clinicians are more conservative in diagnosing BD. Based on the above reasons, we ultimately abandoned converting different scale scores into percentages for correlation analysis, only retained descriptive assessment of symptoms, and indicated the number of respondents.

**Table 1:** Demographic data among patients with three disorders and healthy controls.

Characteristic	Patients (*n* = 310)	HC (*n* = 104)	With two groups (*df* = 412)			SCZ (*n* = 102)	BD (*n* = 103)	MDD (*n* = 105)	With three subgroups (*df* = 307)		
	MD ± SD	MD ± SD	F/χ^2^	*p*	ES(*η* ^2^/V)	MD ± SD	MD ± SD	MD ± SD	F/OR	*p*	ES(*η* ^2^/V)
Age (years)	25^b^ (16–57)	34^b^ (22–45)	121.16	<0.001 ^∗∗^	0.181	29^b^ (16–55)	23^b^ (16–57)	20^b^ (16–43)	14.67	<0.001 ^∗∗^	0.087
Gender (M/F)	115M/195F	104M/0F	90.80	<0.001 ^∗∗^	0.541	53M/49F	34M/69F	28M/77F	15.29^c^	<0.001 ^∗∗^	0.222
Education level (years)	14.0 ± 2.8	13.5 ± 3.0	1.80	0.180	0.004	13.7 ± 2.9	14.4 ± 2.8	13.8 ± 2.6	2.03	0.133	0.013
VFT performance (number)	9.2 ± 4.0	7.5 ± 3.7	15.55	<0.001 ^∗∗^	0.036	8.2 ± 3.6	10.1 ± 4.1	9.5 ± 4.0	6.53	0.002 ^∗^	0.041
Clinical features	—	—	—	—	—	—	—	—	—	—	—
*Age of onset* (*years*)	19^b^ (9–47)	—	—	—	—	22^b^ (13–43)	18^b^ (9–47)	18^b^ (11–43)	4.17	0.016 ^∗^	0.027
*Total duration* (*months*)	52.7 ± 51.4	—	—	—	—	73.5 ± 53.7	60.2 ± 57.3	25.2 ± 24.3	28.87	<0.001 ^∗∗^	0.158
^a^ *PANSS total scores*	73.7 ± 34.7 (*n* = 117)	—	—	—	—	91.0 ± 39.5 (*n* = 48)	65.1 ± 20.5 (*n* = 21)	60.2 ± 26.6 (*n* = 48)	12.28	<0.001 ^∗∗^	0.177
*PANSS-P*	41.2 ± 27.7 (*n* = 117)	—	—	—	—	54.7 ± 32.9 (*n* = 48)	33.6 ± 15.5 (*n* = 21)	31.0 ± 19.5 (*n* = 48)	11.51	<0.001 ^∗∗^	0.168
*PANSS-N*	11.7 ± 4.9 (*n* = 117)	—	—	—	—	13.1 ± 5.4 (*n* = 48)	11.1 ± 3.6 (*n* = 21)	10.6 ± 4.5 (*n* = 48)	3.65	0.029 ^∗^	0.06
*PANSS-G*	20.8 ± 4.5 (*n* = 117)	—	—	—	—	23.2 ± 5.0 (*n* = 48)	18.5 ± 3.0 (*n* = 48)	18.5 ± 3.0 (*n* = 48)	16.37	<0.001 ^∗∗^	0.223
^a^ *YMRS scores*	31.7 ± 16.6 (*n* = 110)	—	—	—	—	35.1 ± 16.8 (*n* = 21)	34.9 ± 17.3 (*n* = 53	24.8 ± 13.5 (*n* = 36)	4.83	0.010 ^∗^	0.083
^a^ *HAMD scores*	23.3 ± 12.5 (*n* = 190)	—	—	—	—	14.3 ± 9.9 (*n* = 32)	21.9 ± 13.0 (*n* = 59)	27.1 ± 11.3 (*n* = 99)	15.09	<0.001 ^∗∗^	0.139
^a^ *HAMA scores*	20.2 ± 11.7 (*n* = 175)	—	—	—	—	13.1 ± 9.9 (*n* = 30)	19.0 ± 11.8 (*n* = 47)	23.0 ± 11.2 (*n* = 98)	9.37	<0.001 ^∗∗^	0.098
Current medications	—	—	—	—	—	—	—	—	—	—	0.182
*Antipsychotics use*, *n* (*%*)	122 (39.4%)	—	—	—	—	81 (79.4%)	40 (38.8%)	1 (1.0%)	133.47^c^	<0.001 ^∗∗^	0.656
*CPZ equivalents* (*mg/day*)	342.8 ± 202.2	—	—	—	—	399.4 ± 206.2	228.1 ± 135.8	—	22.68	<0.001 ^∗∗^	0.16
*Fluoxetine equivalents* (*mg/day*)	26.7 ± 15.5	—	—	—	—	24.4 ± 14.0	20.0 ± 9.0	31.2 ± 17.3	4.84	0.010 ^∗^	0.092
*Mood stabilizers use*, *n* (*%*)	68 (21.9%)	—	—	—	—	3 (2.9%)	53 (51.5%)	12 (11.4%)	80.68	<0.001 ^∗∗^	0.51
Current mood state (BD)	—	—	—	—	—	—	—	—	—	—	—
*Stable*, *n* (*%*)	—	—	—	—	—	—	56 (54.4%)	—	—	—	—
*Mixed*, *n* (*%*)	—	—	—	—	—	—	21 (20.4%)	—	—	—	—
*Manic*, *n* (*%*)	—	—	—	—	—	—	14 (13.6%)	—	—	—	—
*Depressive*, *n* (*%*)	—	—	—	—	—	—	12 (11.7%)	—	—	—	—

*Note:* Data from questionnaires are presented in terms of mean score (M) and standard deviation (SD) in patients and control groups. The *p*‐values were obtained by two‐sample two‐tailed *t*‐test. ES(*η* ^2^/V) indicates effect size, where *η* ^2^ is for continuous variables and *V* is for categorical variables.

Abbreviations: BD, bipolar disorder; HC, healthy control; MDD, major depressive disorder; SCZ, schizophrenia.

^a^Our samples were sourced from outpatient and inpatient departments. For inpatient patients, we used various observer‐rated scales; for outpatient patients, we used self‐rated scales. Some patients, for various reasons, completed these self‐rated scales improperly or even refused to complete them, which resulted in differences in the final sample scales and made correlation analysis impossible. However, in order to present the samples as comprehensively as possible, we chose to describe the scores of the observer‐rated scales and indicate the number of respondents.

^b^Indicates median age.

^c^Indicates *F*‐values for chi‐square test.

^∗^Means *p* < 0.05.

^∗∗^Means *p* < 0.001.

The study was approved by the Human Subjects Protection Committee of West China Hospital (Approval Number 2023‐2157). The participants were recruited from the Mental Health Center, West China Hospital, Sichuan University, between January 2024 and September 2024 (Figure 1).

**Figure 1:**
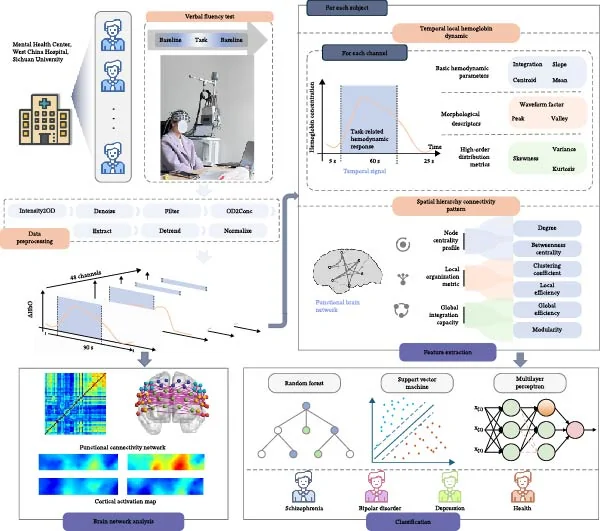
Overview of our proposed method.

The inclusion criteria for patient participants were (1) age range between 16 and 65 years and (2) Han Chinese and fluency in Chinese. Conversely, the exclusion criteria consisted of (1) substance dependence or abuse, (2) severe physical disease or traumatic brain injury, and (3) uncooperative partners.

### 2.2. Measures

#### 2.2.1. Experimental Paradigm

In our study, we employed a modified VFT paradigm (Figure 2A) consisting of three phases: a 30 s pretask period, a 60 s task period, and a 70 s posttask period, which included three subtasks. During the nontask periods, participants repeated a simple counting from 1 to 5 in Chinese. In contrast, the task period required participants to generate phrases using common Chinese characters (e.g., “/hə/(river)”, “/bæ/(white)”, and “/dʌ/(big)”). Each character had a time limit of 20 s. To acquire the fNIRS signal of the objective region, the measurement system primarily focused on the bilateral prefrontal cortex (as shown in Figure 2C), allowing us to examine the neural activity underlying verbal fluency.

**Figure 2:**
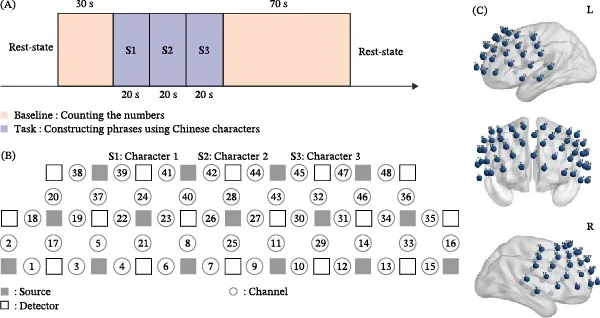
(A) Scheme of verbal fluency task (VFT). (B) The distribution of device probes and channels. (C) Corresponding distribution of channels and brain mapping.

#### 2.2.2. Collection Device

Our study used a near‐infrared optical imaging system (NirScan4000D, Huichuang Corporation, Danyang, China) with a 48 channel setting to record changes in HbO and HbR levels during the VFT. The system had wavelengths of 730, 808, and 850 nm, a sampling rate of 11 Hz, and employed 15 source probe and 16 detector probes with 3 cm separation (Figure 2B).

### 2.3. Material and Method

#### 2.3.1. fNIRS Preprocessing

The raw fNIRS signals were analyzed using the HOMER2 toolbox, and the complete preprocessing pipeline is illustrated in Figure S2. Channel quality was evaluated using the coefficient of variation (CV), with CV > 0.30 indicating artifact‐contaminated channels. Overall quality metrics and the subject‐level distribution of bad channels are reported in Table S1, while the most frequently affected channels and comparisons across diagnostic groups are provided in Table S2; no significant group difference was detected (Kruskal–Wallis *H* = 2.39, *p* = 0.496).

The intensity signals were first converted into optical density and subsequently corrected for temporal correlations using the TDDR method. To mitigate physiological noise arising from respiration, cardiac activity, and other systemic process, a low‐pass filtering (0–0.1 Hz, 3rd order Butterworth) was applied. By applying the modified Beer–Lambert law, the optical signal was transformed into hemoglobin concentration changes (HbO and HbR). The derived hemodynamic data underwent detrending through 1st‐order polynomial fitting, followed by baseline normalization using the mean value of the 5 s pretask period.

#### 2.3.2. Multiscale Spatiotemporal Network Fusion (MSNF)

Single‐scale feature biomarkers often fail to comprehensively reflect the complexity of multichannel fNIRS signal. To highlight different perspectives of the fNIRS signal, in this study, we introduce the MSNF feature method by combining the temporal dynamic and spatial topology. This multiscale approach integrates local hemodynamic dynamics with global network interactions, providing complementary perspectives on neurovascular coupling mechanisms.

To minimize potential interference from baseline fluctuations, the analysis window was defined as 90 s, spanning from 5 s prior to task initiation to 25 s after task completion. Furthermore, we prioritize the HbO signal that is more sensitive to cognitive function. Temporal statistical features were extracted separately for each channel through localized signal analysis. FC matrices constructed using Spearman correlation coefficients were subsequently subjected to graph theoretical analysis to quantify network properties. The detailed extraction procedure is outlined in Supporting Algorithm 1, while the definitions and formulations of the temporal and spatial features used in the MSNF method are summarized in Table 2.

**Table 2:** Formulation of temporal and spatial features in the MSNF method.

Feature	Formula	
Temporal local hemoglobin dynamic *Given the fNIRS signal for a specific channel as* *x*(*t*) ∈ *R* ^*T*^, *where* *t* = 1, 2, …, *T* *denotes the time points during the task period*	Basic hemodynamic parameters	Mean: $µ=\frac{1}{T}\sum_{t=1}^{T}x\left(t\right)$
		Slope: $\text{slope}=\frac{\sum_{i=1}^{n}\left(t_{i}-\bar{t}\right)\left(x_{i}-\bar{x}\right)}{\sum_{i=1}^{n}{\left(t_{i}-\bar{t}\right)}^{2}}$
		Integral: $I=\sum_{t=1}^{T}x\left(t\right)\cdot \Delta t$
		Centroid: $C=\frac{\sum_{t=1}^{T}t\cdot x\left(t\right)}{\sum_{t=1}^{T}x\left(t\right)}$
	Morphological descriptors	Waveform factor: $\text{WF}=\frac{\sqrt{\frac{1}{T}\sum_{t=1}^{T}x{\left(t\right)}^{2}}}{µ}$
		Peak: peak = max(*x*(*t*))
		Valley: valley = min(*x*(*t*))
	High‐order distribution metrics	Variance: $\sigma^{2}=\frac{1}{T}\sum_{t=1}^{T}{\left(x\left(t\right)-\mu \right)}^{2}$
		Skewness: $\text{Skew}=\frac{1}{T}\sum_{t=1}^{T}{\left(\frac{x\left(t\right)-µ}{\sigma }\right)}^{3}$
		Kurtosis: $\text{Kurt}=\frac{1}{T}\sum_{t=1}^{T}{\left(\frac{x\left(t\right)-µ}{\sigma }\right)}^{4}$
Spatial hierarchy connectivity pattern *The FC matrix is thresholded to form an undirected binary graph* *G* = (*V*, *E*)	Node centrality profile	Degree: $\deg\left(v_{i}\right)=\sum_{j=1}^{N}A_{i,j}$
		Betweenness centrality: $\text{BC}\left(vi\right)=\sum_{s\neq i\neq t}\frac{\sigma_{st}\left(v_{i}\right)}{\sigma_{st}}$ *σ*_*s* *t*_ *is the total number of shortest paths from node* *s* *to node* *t*, *and* *σ*_*s* *t*_(*v*_*i*_) *is the number of those paths that pass through node* *v*_*i*_
	Local organization metric	Clustering coefficient: $C\left(v_{i}\right)=\frac{2T_{i}}{k_{i}\left(k_{i}-1\right)}$ *T*_*i*_ *is the number of triangles involving node* *v*_*i*_, *and* *k*_*i*_ *is its degree*
		Local efficiency: $E_{\text{ioc}}\left(v_{i}\right)=\frac{1}{k_{i}\left(k_{i}-1\right)}\sum_{j,h\in N_{i}}\frac{1}{d_{jh}}$
	Global integration capacity	Global efficiency: $E_{\text{glob}}=\frac{1}{N\left(N-1\right)}\sum_{i\neq j}\frac{1}{d_{ij}}$
		Modularity: $Q=\frac{1}{2m}\sum_{i,j}\left[A_{ij}-\frac{k_{i}k_{j}}{2m}\right]\delta \left(c_{i},c_{j}\right)$ *m* *is the total number of edges*, *c*_*i*_ *is the community to which node* *i* *belongs*

##### 2.3.2.1. Temporal Local Hemoglobin Dynamic

Previous studies have identified four clinically relevant temporal statistics for hemodynamic signal analysis: mean, slope, integral, and centroid of HbO. The mean represents signal magnitude during tasks, indicating activation levels in response to stimuli. The slope, derived from linear regression of initial 5 s task signals, reflects response velocity and cognitive agility. The integral quantifies the total area under the hemodynamic curve, representing activation energy expenditure. The centroid denotes the temporal center of mass for hemodynamic activity, characterizing temporal distribution patterns.

These metrics assess neural activation dynamics in clinical proposition. Following this framework, we further explore temporal dynamics metrics to develop a comprehensive feature extraction protocol for quantifying hemodynamic temporal patterns.

The temporal feature extraction protocol comprised three complementary dimensions: (1) basic hemodynamic parameters evaluating response magnitude and trend through temporal integration, linear slope, mean value, and hemodynamic centroid; (2) morphological descriptors quantifying signal shape characteristics via peak amplitudes and waveform factor (RMS/mean ratio); and (3) statistical distribution metrics assessing signal variability, asymmetry, and peakedness using variance, skewness, and kurtosis, respectively. This hierarchical approach ensures comprehensive characterization of both macroscopic hemodynamic patterns and microscopic signal fluctuations.

##### 2.3.2.2. Spatial Hierarchy Connectivity Pattern

Interactions between brain regions offer insights into the mechanisms of psychiatric disorders. Brain connectivity networks, as simplified representations of these interactions, are commonly used for diagnosis and classification. Inspired by FC, we extract graph‐based spatial topological features to characterize hierarchical interactions among brain regions.

For the construction of spatial topological features, we specifically employed the Spearman correlation coefficient to calculate the FC matrices. Unlike linear metrics, this rank‐based measure is robust to nonlinear hemodynamic trends, ensuring that the derived topological features remain stable. The Spearman correlation measures the monotonic relationship intensity of two variables as follows:

Ai,j=1−6∑idi2nn2−1,

where *d* means rank differential of *i* data pair, and *n* represents the total number of observed data.

To extract graph theory features from FC matrices, we filtered weak FC using four sparsity thresholds ranging from 20% to 50%. Subsequently, we computed graph theory features according to the undirected binary graph acquired above.

The graph theoretical analysis systematically evaluated four fundamental network properties: (1) node centrality profiles delineating regional influence through degree (connection quantity) and betweenness centrality (communication control), (2) local organization metrics characterizing neighborhood connectivity using clustering coefficient (triangular connection density) and local efficiency (fault‐tolerant transmission capacity), (3) global integration capacity reflecting whole‐brain communication efficiency via global efficiency (parallel information transfer capability), and (4) modular segregation quantifying functional specialization through modularity index (community structure robustness). This multilevel framework adheres to the canonical network analysis paradigm in neuroimaging.

### 2.4. Classification Model Construction

To comprehensively evaluate the diagnostic performance of the proposed MSNF framework, we employed a diverse set of classifiers representing distinct inductive biases. Specifically, this selection spans three fundamental methodological categories: RF (ensemble learning based on bagging and decision trees), SVM (kernel‐based methods), and MLP (artificial neural networks). RF, SVM, and MLP were selected to assess robustness across different learning paradigms. RF, an ensemble learning method based on bagging and decision trees, was chosen for its inherent robustness to multicollinearity among different feature metrics and its ability to handle heterogeneous feature scales, making it particularly suitable for neuroimaging datasets with moderate sample sizes. SVM is particularly robust for small sample sizes and excels at finding optimal hyperplanes in high‐dimensional spaces. MLP was utilized to capture latent nonlinear interactions between the fused temporal dynamics and spatial topological features.

To further broaden the evaluation and benchmark the proposed method, we incorporated eXtreme Gradient Boosting (XGBoost) and a one‐dimensional convolutional neural network (1D‐CNN), which is specifically designed for the fNIRS paradigm. XGBoost was employed to evaluate the performance of gradient‐based boosting algorithms, which sequentially correct errors from previous predictors. Additionally, we implemented a 1D‐CNN following the research [37]. Unlike the fully connected architecture of MLP, the 1D‐CNN applies convolutional filters to the feature vectors. This approach allows for the extraction of local patterns and dependencies within the structured feature set, providing a distinct deep learning perspective to validate the discriminative power of the constructed features.

To minimize overfitting and ensure the reliability of model evaluation given the limited sample size, we employed a nested cross‐validation (NCV) framework. Stratified sampling was applied to maintain the ratio of diagnostic classes across folds, and data partitioning was strictly performed at the subject level to prevent data leakage, as each participant contributed a single aggregated feature vector. Crucially, hyperparameter optimization was standardized across all classifiers to ensure a fair comparison. The NCV consists of two nested loops: an outer loop (10‐fold) to assess generalization performance on unseen test data and an inner loop (5‐fold) dedicated to hyperparameter tuning. Within the inner loop, we conducted a systematic grid search for each classifier to identify the optimal configuration that maximized validation accuracy. This standardized optimization protocol ensures that observed performance differences are attributable to the intrinsic capabilities of the models rather than inconsistencies in hyperparameter tuning.

To evaluate the comprehensive performance of each model, the commonly used indicators, accuracy (ACC), F1 score (F1), recall, and area of the curve (AUC), were used. For binary classification, we calculated the indicators as follows:

Precision=TPTP+FP,

Recall=TPTP+FN,

FPR=FPFP+TN,

ACC=TP+TNTP+TN+FP+FN,

F1=2Precision⋅RecallPrecision+Recall,

AUC=∑i=1n−1FPRi−FPRi−1TPRi−FPRi2.

For multiclass classification, we utilized a macroaveraging method to represent the classification metrics for each category. These metrics included ACC_Macro_, F1_Macro_, Recall_Macro_, and AUC_Macro_. The formulas are as follows:

ACCMacro=1N∑iNACCi,

F1Macro=1N∑iNF1i,

RecallMacro=1N∑iNRecalli,

AUCMacro=1N∑iNAUCi.

### 2.5. FC Analysis

Statistical analysis and visualization were conducted using the NirSpark software package and BrainNet View software package. Distinct from the graph‐based feature extraction described above, for the direct statistical comparison of FC strength between groups, we employed the Pearson correlation coefficient. This approach sensitively captures the linear magnitude of coupling. The resulting correlation coefficients were subsequently normalized using the Fisher *z*‐transformation to satisfy the normality assumption required for parametric testing.

FC strength (Fisher’s *z*‐transformed correlation coefficients) was compared across the four groups using a one‐way analysis of variance (ANOVA) for each region of interest (ROI)‐to‐ROI link. To address the multiple comparison problem inherent in evaluating multiple connectivity links, the *p*‐values from the ANOVA were corrected using the false discovery rate (FDR) method (Benjamini–Hochberg procedure). For links exhibiting significant group differences after correction (*p*_FDR_ < 0.05), post hoc pairwise comparisons were conducted using Welch’s *t*‐tests to handle potential inequalities in variance. These post hoc *p*‐values were also FDR‐corrected to confirm specific group differences. Effect sizes were evaluated using *Cohen’s d* for pairwise comparisons.

## 3. Results

### 3.1. Four‐Class Classification Results for Multidisease and HC Groups

This subsection delineates the hierarchical discriminative capacity of MSNF features in complex psychiatric nosology, specifically addressing four‐way classification challenges encompassing SCZ, BD, MDD, and healthy populations.

As synthesized in Table 3, multimodal integration substantially elevates computational diagnostic precision in quadripartite classification frameworks. Initial baseline performance for SVM yielded an AUC of 0.81 ± 0.04, which surpassed baseline limitations to reach 0.86 ± 0.02 following MSNF integration. The RF model demonstrated enhanced discriminative capacity, achieving 0.87 ± 0.02 AUC with MSNF (baseline: 0.84 ± 0.03), while MLP performance improved from 0.76 ± 0.05 to 0.81 ± 0.03. For XGBoost, baseline performance achieved an AUC of 0.79 ± 0.04, which increased to 0.83 ± 0.03 after MSNF integration. Similarly, the 1D‐CNN exhibited improved discriminative performance, with AUC rising from 0.80 ± 0.05 to 0.83 ± 0.04. These advancements primarily originate from MSNF’s neurotopological characterization, which refines classification boundaries through quantification of functional network dynamic integration capacity across key neural circuits.

**Table 3:** Results of cross‐diagnostic classification of psychiatric disorders.

Type	Feature	Method	Accuracy	Recall (macro)	F1 (macro)	AUC (OvR)
SCZ/BD/MDD/HC	Baseline	SVM	0.56 ± 0.07	0.56 ± 0.07	0.55 ± 0.08	0.81 ± 0.04
		RF	0.60 ± 0.07	0.60 ± 0.07	0.59 ± 0.07	0.84 ± 0.03
		MLP	0.51 ± 0.05	0.48 ± 0.08	0.51 ± 0.05	0.76 ± 0.05
		XGBoost	0.53 ± 0.06	0.53 ± 0.07	0.53 ± 0.07	0.79 ± 0.04
		1D‐CNN	0.50 ± 0.04	0.51 ± 0.04	0.49 ± 0.04	0.80 ± 0.05
	Ours	SVM	**0.60 ± 0.07**	0.60 ± 0.05	0.59 ± 0.07	**0.86 ± 0.02**
		RF	**0.64 ± 0.06**	0.64 ± 0.06	0.63 ± 0.06	**0.87 ± 0.02**
		MLP	**0.58 ± 0.06**	0.58 ± 0.06	0.57 ± 0.06	**0.81 ± 0.03**
		XGBoost	**0.59 ± 0.09**	0.59 ± 0.09	0.58 ± 0.09	**0.83 ± 0.03**
		1D‐CNN	**0.55 ± 0.04**	0.55 ± 0.04	0.54 ± 0.06	**0.83 ± 0.04**

*Note*: Bold indicates the results of the proposed method that outperform the corresponding baseline methods.

The diagnostic superiority of MSNF is visually substantiated by Figure 3. Comparative evaluation reveals that MSNF‐driven classifiers (Figure 3b) exhibit expanded high‐confidence prediction regions with an AUC of 0.88 in quadripartite classification, markedly outperforming baseline features (AUC = 0.83, Figure 3a). This progression underscores MSNF’s critical role in disentangling multidimensional neuropathological signatures across psychiatric spectra.

**Figure 3:**
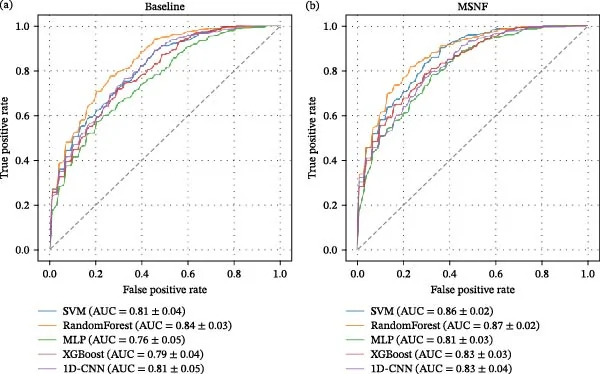
ROC curves comparing classification performance (AUC values) of baseline features versus MSNF in cross‐diagnostic classification of psychiatric disorders, baseline features (a) and MSNF (b).

Importantly, the advantages of MSNF extend beyond improved prediction accuracy, offering a deeper characterization of the dynamic features of neural networks in psychiatric disorders. Its unique multiscale fusion mechanism refines cross‐disease functional network patterns, enhancing neural circuit integrity and classification boundary discernibility. This capability helps address the limitations of traditional methods in multidisease differentiation, providing strong support for future precision diagnostic technologies. Further research could explore the broader applicability of MSNF across diverse populations and its potential in personalized interventions and precision medicine, driving advancements in computational psychiatric diagnostics.

### 3.2. Classification Across Three Psychiatric Conditions and HCs Using MSNF Features

This subsection systematically evaluates the diagnostic potential of MSNF features in tripartite classification frameworks, with specific focus on delineating neurobiological boundaries among SCZ, BD, MDD, and HCs. Table 4 shows the triclassification results for SCZ, BD, depression and health, and the corresponding receiver operating characteristic (ROC) curves are shown in Figure 4a–h.

Figure 4ROC curves comparing classification performance (AUC values) of baseline features versus MSNF in discriminating psychiatric disorders and health controls, baseline features (a–d) and MSNF (e–h). (a,e) Comparison among SCZ, HC, and BD. (b,f) Comparison among MDD, HC, and SCZ. (c,g) Comparison among MDD, HC, and BD. (d,h) Comparison among SCZ, MDD, and BD.

**Figure:**
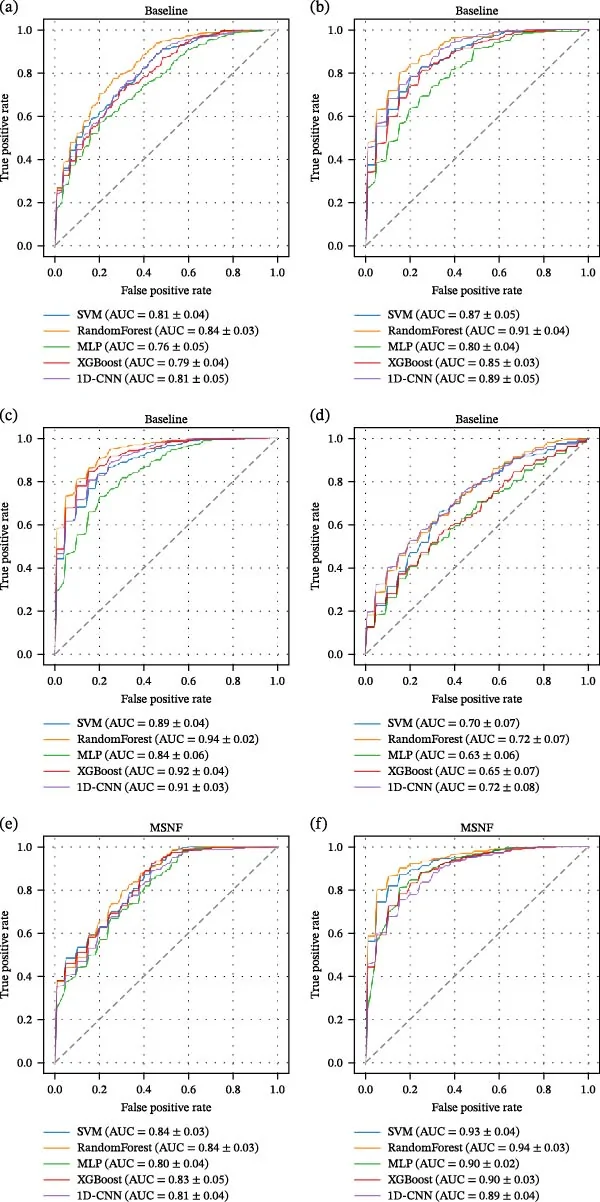

**Figure:**
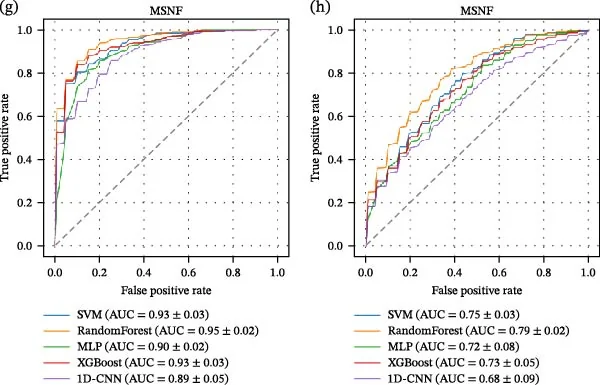

**Table 4:** Classification results across three psychiatric conditions and healthy controls.

Type	Feature	Method	Accuracy	Recall (macro)	F1 (macro)	AUC (OvR)
SCZ/HC/BD	Baseline	SVM	0.66 ± 0.06	0.66 ± 0.06	0.66 ± 0.07	0.82 ± 0.03
		RF	0.62 ± 0.07	0.62 ± 0.07	0.61 ± 0.07	0.82 ± 0.04
		MLP	0.59 ± 0.08	0.59 ± 0.08	0.57 ± 0.08	0.79 ± 0.05
		XGBoost	0.58 ± 0.08	0.58 ± 0.08	0.57 ± 0.08	0.80 ± 0.04
		1D‐CNN	0.65 ± 0.08	0.65 ± 0.08	0.63 ± 0.09	0.83 ± 0.04
	Ours	SVM	0.60 ± 0.07	0.60 ± 0.07	0.59 ± 0.07	**0.84 ± 0.03**
		RF	**0.65 ± 0.08**	0.64 ± 0.08	0.63 ± 0.08	**0.84 ± 0.03**
		MLP	0.62 ± 0.07	0.62 ± 0.07	0.62 ± 0.06	0.80 ± 0.04
		XGBoost	**0.63 ± 0.08**	**0.63 ± 0.08**	**0.62 ± 0.08**	**0.83 ± 0.05**
		1D‐CNN	0.61 ± 0.07	0.61 ± 0.07	0.59 ± 0.07	0.81 ± 0.04
MDD/HC/SCZ	Baseline	SVM	0.69 ± 0.08	0.70 ± 0.08	0.69 ± 0.08	0.87 ± 0.05
		RF	0.77 ± 0.07	0.77 ± 0.07	0.77 ± 0.07	0.91 ± 0.04
		MLP	0.64 ± 0.05	0.64 ± 0.05	0.63 ± 0.05	0.80 ± 0.04
		XGBoost	0.69 ± 0.03	0.69 ± 0.03	0.68 ± 0.03	0.85 ± 0.03
		1D‐CNN	0.70 ± 0.09	0.70 ± 0.09	0.69 ± 0.10	0.89 ± 0.05
	Ours	SVM	**0.81 ± 0.07**	0.82 ± 0.07	0.81 ± 0.07	**0.93 ± 0.04**
		RF	**0.83 ± 0.06**	0.83 ± 0.06	0.83 ± 0.07	**0.94 ± 0.03**
		MLP	**0.76 ± 0.06**	0.76 ± 0.06	0.76 ± 0.06	**0.90 ± 0.02**
		XGBoost	**0.74 ± 0.05**	0.74 ± 0.05	0.73 ± 0.06	**0.90 ± 0.03**
		1D‐CNN	**0.72 ± 0.06**	0.72 ± 0.06	0.71 ± 0.07	**0.89 ± 0.04**
MDD/HC/BD	Baseline	SVM	0.72 ± 0.06	0.72 ± 0.06	0.72 ± 0.07	0.89 ± 0.04
		RF	0.81 ± 0.05	0.81 ± 0.05	0.81 ± 0.05	0.94 ± 0.02
		MLP	0.69 ± 0.07	0.69 ± 0.08	0.69 ± 0.08	0.84 ± 0.06
		XGBoost	0.79 ± 0.08	0.79 ± 0.08	0.78 ± 0.09	0.92 ± 0.04
		1D‐CNN	0.74 ± 0.04	0.74 ± 0.04	0.73 ± 0.04	0.91 ± 0.03
	Ours	SVM	**0.80 ± 0.06**	0.80 ± 0.06	0.80 ± 0.06	**0.93 ± 0.03**
		RF	**0.83 ± 0.07**	0.83 ± 0.07	0.83 ± 0.06	**0.95 ± 0.02**
		MLP	**0.78 ± 0.03**	0.78 ± 0.03	0.77 ± 0.03	**0.90 ± 0.02**
		XGBoost	**0.80 ± 0.06**	0.80 ± 0.06	0.80 ± 0.07	**0.93 ± 0.03**
		1D‐CNN	0.72 ± 0.06	0.72 ± 0.07	0.71 ± 0.07	0.89 ± 0.05
SCZ/MDD/BD	Baseline	SVM	0.50 ± 0.09	0.50 ± 0.09	0.50 ± 0.09	0.70 ± 0.07
		RF	0.52 ± 0.10	0.52 ± 0.10	0.51 ± 0.10	0.72 ± 0.07
		MLP	0.47 ± 0.10	0.47 ± 0.10	0.47 ± 0.10	0.63 ± 0.06
		XGBoost	0.46 ± 0.05	0.46 ± 0.05	0.45 ± 0.05	0.65 ± 0.07
		1D‐CNN	0.53 ± 0.09	0.53 ± 0.10	0.53 ± 0.09	0.72 ± 0.08
	Ours	SVM	**0.54 ± 0.06**	0.54 ± 0.06	0.53 ± 0.06	**0.75 ± 0.03**
		RF	**0.59 ± 0.03**	0.59 ± 0.03	0.58 ± 0.04	**0.79 ± 0.02**
		MLP	**0.51 ± 0.11**	0.51 ± 0.11	0.51 ± 0.11	**0.72 ± 0.08**
		XGBoost	**0.54 ± 0.08**	0.53 ± 0.08	0.53 ± 0.08	**0.73 ± 0.05**
		1D‐CNN	0.50 ± 0.08	0.50 ± 0.08	0.48 ± 0.08	0.68 ± 0.09

*Note*: Bold indicates the results of the proposed method that outperform the corresponding baseline methods.

In the classification task of SCZ/healthy/BD, the AUC value of the RF model reaches 0.84 ± 0.03, which is 0.02 higher than the baseline (baseline 0.82 ± 0.04), but the overall improvement is not obvious, and the accuracy is only about 0.60, which corresponds to the previous binary classification results of SCZ and BD (Figure 4a,e). Heterogeneity among diseases has a decisive impact on classification efficiency. In the classification tasks of depression/health/SCZ and depression/health/BD, the AUC values of the three models using MSNF are further increased to more than 0.90, which is a great improvement compared with the baseline (Figure 4b,f and Figure 4c,g, respectively). The key is that local efficiency and betweenness centrality quantify the difference in information transmission efficiency of key brain regions. The classification boundaries between SCZ and depression and between BD and depression were optimized. In the pure disease three‐classification task, the AUC value of the RF model after using MSNF is 0.79 ± 0.02, which is 0.07 higher than the baseline (baseline 0.72 ± 0.07), indicating the effectiveness of MSNF (Figure 4d,h).

### 3.3. Effectiveness of MSNF‐Based ML Models in Differentiating Psychiatric Disorders

This section evaluates the efficacy of ML models combined with MSNF features in differentiating three disorders. Table 5 shows the binary classification results for SCZ, BD, and depression. The corresponding ROC curves obtained using the baseline and MSNF features are presented in Figure 5a,c and Figure 5d,f, respectively.

**Figure 5:**
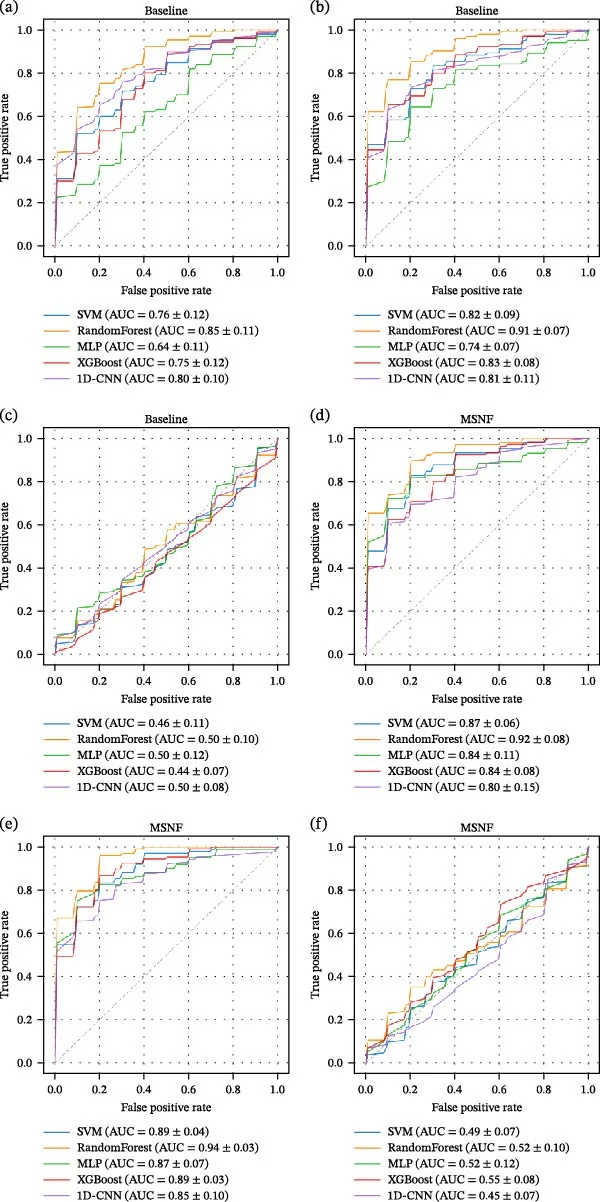
ROC curves comparing classification performance (AUC values) of baseline features versus MSNF in discriminating psychiatric disorders, baseline features (a–c) and MSNF (d–f). (a,d) Comparison between SCZ and MDD. (b,e) Comparison between BD and MDD. (c,f) Comparison between SCZ and BD.

**Table 5:** Classification results of MSNF‐based machine learning models in differentiating psychiatric disorders.

Type	Feature	Method	Accuracy	Recall	F1‐score	AUC
SCZ/MDD	Baseline	SVM	0.68 ± 0.11	0.68 ± 0.11	0.68 ± 0.11	0.76 ± 0.12
		RF	0.77 ± 0.07	0.77 ± 0.07	0.76 ± 0.07	0.85 ± 0.11
		MLP	0.58 ± 0.09	0.58 ± 0.09	0.57 ± 0.09	0.64 ± 0.11
		XGBoost	0.66 ± 0.08	0.66 ± 0.08	0.66 ± 0.08	0.75 ± 0.12
		1D‐CNN	0.74 ± 0.10	0.74 ± 0.10	0.74 ± 0.10	0.80 ± 0.10
	Ours	SVM	**0.79 ± 0.07**	0.79 ± 0.07	0.79 ± 0.07	**0.87 ± 0.06**
		RF	**0.84 ± 0.08**	0.84 ± 0.08	0.84 ± 0.08	**0.92 ± 0.08**
		MLP	**0.78 ± 0.09**	0.78 ± 0.09	0.78 ± 0.09	**0.84 ± 0.11**
		XGBoost	**0.75 ± 0.12**	0.75 ± 0.11	0.74 ± 0.11	**0.84 ± 0.08**
		1D‐CNN	0.74 ± 0.14	0.74 ± 0.14	0.73 ± 0.14	0.80 ± 0.15
BD/MDD	Baseline	SVM	0.75 ± 0.07	0.76 ± 0.07	0.75 ± 0.07	0.83 ± 0.09
		RF	0.82 ± 0.07	0.82 ± 0.07	0.82 ± 0.07	0.91 ± 0.07
		MLP	0.71 ± 0.08	0.71 ± 0.08	0.71 ± 0.08	0.74 ± 0.07
		XGBoost	0.76 ± 0.08	0.76 ± 0.07	0.76 ± 0.07	0.83 ± 0.08
		1D‐CNN	0.75 ± 0.09	0.75 ± 0.09	0.75 ± 0.09	0.81 ± 0.11
	Ours	SVM	**0.80 ± 0.06**	0.80 ± 0.06	0.80 ± 0.06	**0.89 ± 0.04**
		RF	**0.86 ± 0.06**	0.86 ± 0.06	0.86 ± 0.06	**0.94 ± 0.03**
		MLP	**0.80 ± 0.07**	0.80 ± 0.08	0.79 ± 0.08	**0.87 ± 0.07**
		XGBoost	**0.83 ± 0.04**	0.83 ± 0.04	0.83 ± 0.04	**0.89 ± 0.03**
		1D‐CNN	0.74 ± 0.07	0.74 ± 0.07	0.73 ± 0.07	**0.85 ± 0.10**
SCZ/BD	Baseline	SVM	0.52 ± 0.08	0.52 ± 0.08	0.52 ± 0.08	0.46 ± 0.11
		RF	0.54 ± 0.07	0.54 ± 0.07	0.54 ± 0.07	0.50 ± 0.10
		MLP	0.50 ± 0.09	0.49 ± 0.09	0.46 ± 0.10	0.50 ± 0.12
		XGBoost	0.49 ± 0.08	0.49 ± 0.08	0.47 ± 0.09	0.44 ± 0.07
		1D‐CNN	0.50 ± 0.09	0.50 ± 0.09	0.43 ± 0.11	0.50 ± 0.08
	Ours	SVM	0.43 ± 0.12	0.44 ± 0.08	0.42 ± 0.08	**0.50 ± 0.07**
		RF	0.50 ± 0.09	0.50 ± 0.09	0.49 ± 0.09	**0.52 ± 0.10**
		MLP	**0.54 ± 0.11**	0.53 ± 0.11	0.46 ± 0.14	**0.52 ± 0.12**
		XGBoost	**0.54 ± 0.10**	0.54 ± 0.10	0.52 ± 0.11	**0.55 ± 0.08**
		1D‐CNN	0.44 ± 0.09	0.44 ± 0.09	0.40 ± 0.08	0.45 ± 0.07

*Note*: Bold indicates the results of the proposed method that outperform the corresponding baseline methods.

In the classification task of SCZ and depression, the AUC value of the RF model increased to 0.92 ± 0.08 after using MSNF (baseline: 0.85 ± 0.11), as shown in Figure 5a,d. In the classification task of BD and depression, the AUC value of the RF model combined with MSNF reaches 0.94 ± 0.03 (baseline: 0.91 ± 0.07), as shown in Figure 5b,e. This improvement is mainly attributed to the contribution of second‐order network features in MSNF, which effectively distinguish the mood swings of BD from the persistent affective inhibition patterns of MDD by quantifying the difference in connectivity efficiency between the default mode network and the limbic system. However, in the classification task of BD and SCZ, the existing features (including baseline features and MSNF) fail to achieve effective discrimination. Under the baseline features, the AUC values of SVM, RF, MLP, XGBoost, and 1D‐CNN models were 0.46 ± 0.11, 0.50 ± 0.10, 0.50 ± 0.12, 0.44 ± 0.07, and 0.50 ± 0.08, respectively, while the AUC values of MSNF only slightly increased to 0.50 ± 0.07 (SVM), 0.52 ± 0.10 (RF), 0.52 ± 0.12 (MLP), and 0.55 ± 0.08 (XGBoost). In contrast, the 1D‐CNN exhibited performance degradation after MSNF integration, with the AUC decreasing to 0.45 ± 0.07. The overall performance is close to random (AUC ≈ 0.5). This result indicates that BD and SCZ exhibit a significant overlap in pathological mechanisms, such as abnormal default mode network FC, making it challenging for existing features to capture their distinct differences. The ROC curves in Figure 5c,f further show that the curves for the classification task of BD and SCZ (AUC ≈ 0.5) are approximately diagonal, which is significantly different from the high separation between BD and depression classification (AUC = 0.94) and SCZ and depression classification (AUC = 0.92). It highlights the decisive influence of heterogeneity among diseases on classification efficiency.

### 3.4. Multiscale Network Features Improve Psychiatric Disorder vs. HC Classification

This section evaluates the performance of optimized ML models in distinguishing psychiatric disorders (SCZ, BD, and depression) from HCs through MSNF features. As summarized in Table 6, feature selection strategies and classification outcomes are presented for SCZ vs. HCs, BD vs. HCs, and depression vs. HCs, with Figure 6a–f providing a comparative visualization of ROC curves. Specifically, Figure 6a–c presents the results obtained using the baseline features, while Figure 6d–f presents the corresponding results obtained using MSNF.

**Figure 6:**
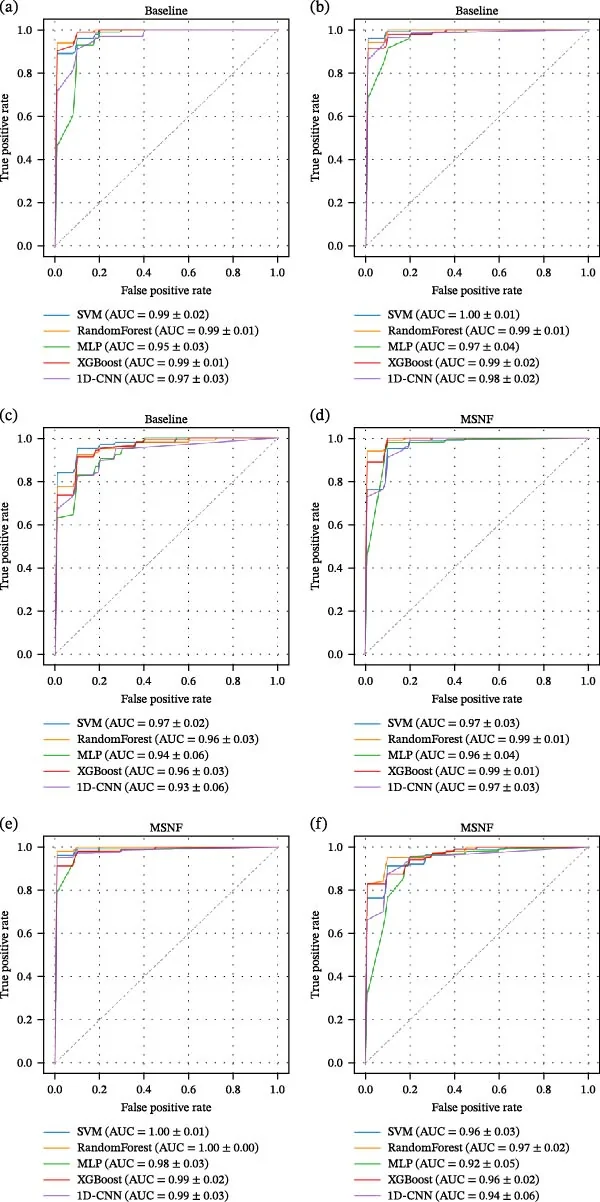
ROC curves comparing classification performance (AUC values) of baseline features versus MSNF in discriminating psychiatric disorders from healthy controls, baseline features (a–c) and MSNF (d–f). (a,d) Comparison between SCZ and HC. (b,e) Comparison between BD and HC. (c,f) Comparison between MDD and HC.

**Table 6:** Classification metrics comparison: baseline features versus MSNF for psychiatric disorders versus healthy controls.

Type	Feature	Method	Accuracy	Recall	F1‐score	AUC
SCZ/HC	Baseline	SVM	0.93 ± 0.05	0.93 ± 0.04	0.93 ± 0.05	0.99 ± 0.02
		RF	0.94 ± 0.05	0.94 ± 0.06	0.94 ± 0.06	0.99 ± 0.01
		MLP	0.89 ± 0.04	0.89 ± 0.04	0.89 ± 0.04	0.95 ± 0.03
		XGBoost	0.93 ± 0.06	0.93 ± 0.06	0.93 ± 0.06	0.99 ± 0.01
		1D‐CNN	0.90 ± 0.05	0.90 ± 0.05	0.90 ± 0.05	0.97 ± 0.03
	Ours	SVM	0.92 ± 0.05	0.92 ± 0.05	0.92 ± 0.05	0.97 ± 0.03
		RF	0.93 ± 0.06	0.93 ± 0.06	0.93 ± 0.06	0.99 ± 0.01
		MLP	**0.94 ± 0.02**	0.94 ± 0.02	0.94 ± 0.02	**0.96 ± 0.04**
		XGBoost	**0.96 ± 0.03**	0.96 ± 0.03	0.96 ± 0.03	**0.99 ± 0.01**
		1D‐CNN	**0.91 ± 0.05**	0.91 ± 0.05	0.91 ± 0.05	**0.97 ± 0.03**
BD/HC	Baseline	SVM	0.95 ± 0.05	0.95 ± 0.05	0.95 ± 0.05	1.00 ± 0.01
		RF	0.96 ± 0.04	0.96 ± 0.04	0.96 ± 0.04	0.99 ± 0.01
		MLP	0.93 ± 0.04	0.93 ± 0.04	0.93 ± 0.04	0.97 ± 0.04
		XGBoost	0.92 ± 0.05	0.92 ± 0.05	0.92 ± 0.06	0.99 ± 0.02
		1D‐CNN	0.95 ± 0.04	0.95 ± 0.04	0.95 ± 0.04	0.98 ± 0.02
	Ours	SVM	0.94 ± 0.04	0.94 ± 0.04	0.94 ± 0.04	1.00 ± 0.01
		RF	**0.96 ± 0.03**	0.96 ± 0.03	0.96 ± 0.03	**1.00 ± 0.0**
		MLP	**0.96 ± 0.04**	0.96 ± 0.04	0.96 ± 0.04	**0.98 ± 0.03**
		XGBoost	**0.95 ± 0.04**	0.95 ± 0.04	0.95 ± 0.04	0.99 ± 0.02
		1D‐CNN	**0.96 ± 0.04**	0.96 ± 0.04	0.96 ± 0.04	**0.99 ± 0.03**
MDD/HC	Baseline	SVM	0.90 ± 0.04	0.9 ± 0.04	0.90 ± 0.05	0.97 ± 0.02
		RF	0.90 ± 0.05	0.90 ± 0.05	0.90 ± 0.05	0.96 ± 0.03
		MLP	0.84 ± 0.07	0.84 ± 0.07	0.84 ± 0.07	0.94 ± 0.06
		XGBoost	0.89 ± 0.05	0.89 ± 0.05	0.88 ± 0.05	0.96 ± 0.03
		1D‐CNN	0.86 ± 0.08	0.86 ± 0.08	0.86 ± 0.08	0.93 ± 0.06
	Ours	SVM	**0.91 ± 0.07**	0.91 ± 0.07	0.91 ± 0.07	0.96 ± 0.03
		RF	**0.91 ± 0.06**	0.92 ± 0.06	0.91 ± 0.06	**0.97 ± 0.02**
		MLP	**0.86 ± 0.08**	0.86 ± 0.08	0.86 ± 0.09	0.92 ± 0.05
		XGBoost	0.87 ± 0.08	0.87 ± 0.07	0.87 ± 0.08	**0.96 ± 0.02**
		1D‐CNN	**0.90 ± 0.06**	0.90 ± 0.06	0.90 ± 0.06	**0.94 ± 0.06**

*Note*: Bold indicates the results of the proposed method that outperform the corresponding baseline methods.

For SCZ classification, the MLP model utilizing multimodal synergistic network features achieved an AUC of 0.96 ± 0.04, demonstrating a 0.01 improvement over baseline features (AUC: 0.95 ± 0.03), as shown in Figure 6a,d. In BD classification, models incorporating MSNF matched the baseline AUC performance while exhibiting reduced standard deviation (SD), suggesting enhanced robustness across repeated cross‐validation trials (Figure 6b,e). The strong congruence between BD pathology and VFT biomarkers likely contributed to the comparable performance of both approaches. In depression classification, models with MSNF achieved slightly higher overall accuracy (up to 0.91 ± 0.07) than baseline features, indicating that MSNF can better capture discriminative information for MDD diagnosis. The corresponding ROC curves are shown in Figure 6c,f. Second‐order topological features quantifying local network efficiency emerged as particularly discriminative for capturing depression‐related neuropathological heterogeneity. Crucially, MSNF’s first‐order temporal features effectively captured regional signal variability, while second‐order graph‐theoretical features enhanced model interpretability by encoding functional network topology, thereby improving recognition of complex disease patterns.

### 3.5. Statistical Validation of Classification Performance

We validated the statistical significance of our diagnostic accuracies using permutation testing (1000 iterations), ensuring the results were not artifacts of random chance. We further quantified the magnitude of this significance by calculating *Z*‐scores shown in Figure 7. While all comparisons involving HC and the differentiation of MDD from other psychiatric conditions achieved high statistical significance with *Z*‐scores surpassing 8.0, the BD versus SCZ task exhibited negligible deviation from the null distribution (*Z* < 1.0), as further demonstrated by the overlapping distributions in Figures S3d and S4d. Rather than a methodological failure, this latter finding serves as a statistical validation of the intrinsic phenomensological and hemodynamic similarities characterizing these two psychotic spectrum disorders. For detailed observed‐versus‐null performance distributions, refer to the box plots in Figure S3a–f and the permutation histograms in Figure S4a–f.

**Figure 7:**
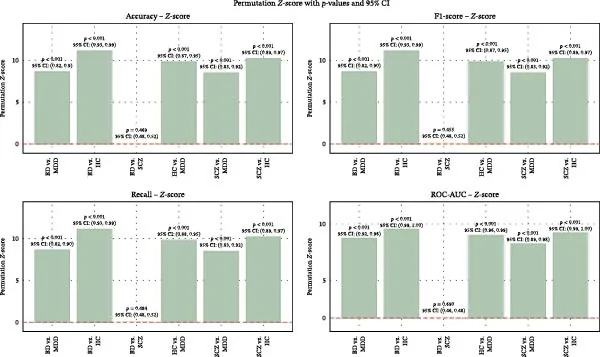
Random forest performance expressed as permutation *Z*‐scores. The *Z*‐score represents the number of standard deviations by which the observed accuracy exceeds the random null mean. Green bars (*Z* > 3) indicate performance significantly above chance (approx. *p* < 0.001), whereas the low *Z*‐score for BD vs. SCZ (beige) reflects the difficulty in distinguishing these conditions.

### 3.6. Sensitivity Analysis

To investigate whether the classification performance was driven by demographic differences rather than disease‐specific neurophysiological patterns, we performed a sensitivity analysis by adjusting for covariates (age and gender), as shown in Figure 8a–f. We employed a regress‐out strategy to remove the variance associated with these covariates from the fNIRS features before classification.

**Figure 8:**
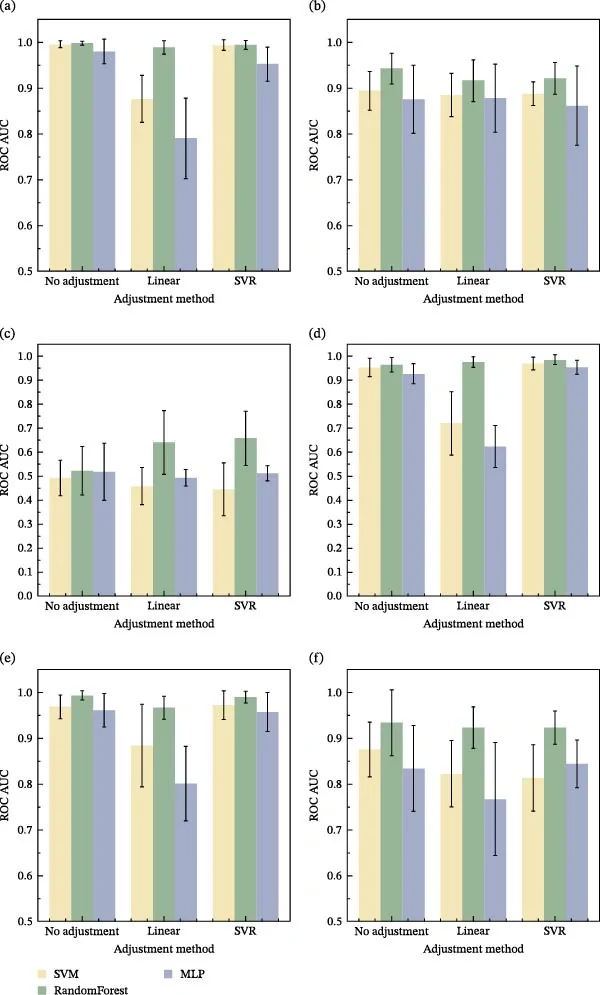
Sensitivity analysis of covariate adjustment (age and sex) on model performance. The bar charts display ROC AUC scores across six binary classification tasks. Performance was evaluated using original features (no adjustment) versus features adjusted via linear regression (linear) or support vector regression (SVR) within the cross‐validation loop. Error bars represent standard deviations. (a) Comparison between BD and HC. (b) Comparison between BD and MDD. (c) Comparison between BD and SCZ. (d) Comparison between HC and MDD. (e) Comparison between HC and SCZ. (f) Comparison between SCZ and MDD.

Specifically, for each feature, we modeled its relationship with age and gender and used the resulting residuals as the adjusted feature inputs. This process was strictly conducted within a NCV to prevent data leakage, meaning the regression models were fitted solely on the training data and then applied to the test data. We evaluated the robustness of our method using three experimental conditions including a baseline without adjustment, a linear regression–based adjustment, and a nonlinear adjustment using support vector regression (SVR). Consistency across experiments was examined by applying three independent classifiers to all pairwise tasks, as shown in Figure 8.

Sensitivity analysis confirmed the robustness of our proposed features against demographic confounding. As shown in Figure 8a–f, classification performance remained stable across pairwise tasks regardless of whether age and gender were adjusted using linear or nonlinear methods. The features maintained high discriminative power even after rigorous covariate removal, indicating that the diagnostic accuracy is driven by intrinsic neurophysiological markers specific to the disorders rather than demographic disparities between groups.

We performed a one‐way analysis of covariance (ANCOVA) on 768 extracted features, comprising both hemodynamic indices and graph topological metrics, to evaluate differences across diagnostic groups (BD, MDD, SCZ, and HC). Age and gender were included as covariates to control for demographic heterogeneity, with all *p*‐values adjusted using the Benjamini–Hochberg FDR correction (*q* < 0.05).

As shown in Figure 9, the analysis revealed widespread group differences. The Manhattan plot (Figure 9a) indicates that 645 biomarkers (84.0%) survived correction, often yielding −log_10_⁡(*p*) values exceeding 30. This pattern implies pervasive alterations in cortical function and network organization associated with these disorders. The raw *p*‐value distribution (Figure 9b) exhibits a pronounced peak near zero, further confirming the presence of robust biological signals. Importantly, a comparison of the top 20 discriminative biomarkers (Figure 9c) demonstrates that the group effect vastly outweighs age and gender contributions. The distributions of these biomarkers across the four diagnostic groups are further visualized in Figure S5. These results confirm that the identified biomarkers reflect the underlying pathophysiology rather than demographic confounds. Male‐only subgroup analyses further assessed sex‐related confounding. Performance across six pairwise tasks and three adjustment strategies is reported in Table S3, with ROC curves and sensitivity results in Figures S6a–f and S7a–f. Results were consistent with the full cohort except for the MLP model in the BD vs. MDD task (*p* = 0.049; Table S4); feature‐level findings are summarized in Figure S8a, b.

**Figure 9:**
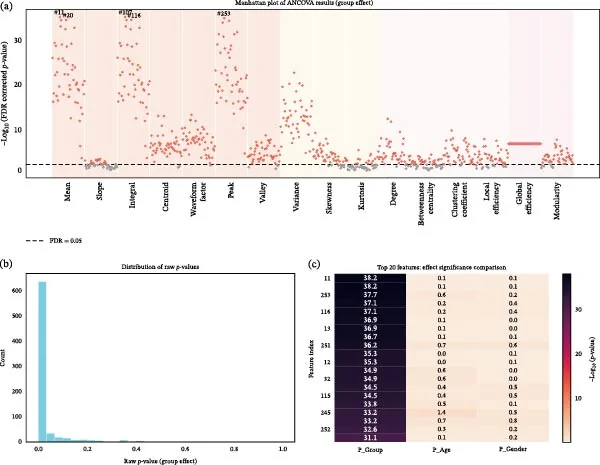
Statistical evaluation of group differences across extracted features. (a) Manhattan plot displaying the negative log‐transformed FDR‐corrected *p*‐values for all 768 features. Red dots indicate significant biomarkers surviving the threshold (dashed line, FDR = 0.05), revealing widespread group effects. (b) Histogram of raw *p*‐values exhibiting a pronounced skew toward zero, confirming the presence of robust biological signals. (c) Heatmap comparing the statistical significance of the group effect against age and gender covariates for the top 20 discriminative features, demonstrating that observed differences are driven by the diagnostic group rather than demographic factors.

### 3.7. ROI‐Based FC Across Groups

The fronto‐temporal regions were divided into eight functionally relevant ROIs, including left/right dorsolateral prefrontal cortex (dlPFC_L and dlPFC_R), left/right ventrolateral prefrontal cortex (vlPFC_L and vlPFC_R), left/right superior temporal gyrus (STC_L and STC_R), and the dorsolateral/ventrolateral frontal pole cortex (DFPC and VFPC). These brain areas have been widely recognized in previous studies [38] as being closely related to psychological functions such as emotional regulation and cognitive control. Through this refined parcellation, a deeper understanding of disease‐related FC patterns was sought, aiming to reveal the neurobiological commonalities and specificities across disorders.

To visually present the overall connectivity strength between these eight brain regions for the four participant groups, average FC matrices were computed for each group, and heatmaps along with 3D brain region–based connectivity plots were generated (Figure 10). The results indicated that the HC group exhibited higher FC strength between most brain regions, with a denser distribution of connections, whereas the mental disorder groups showed overall weakened connectivity.

**Figure 10:**
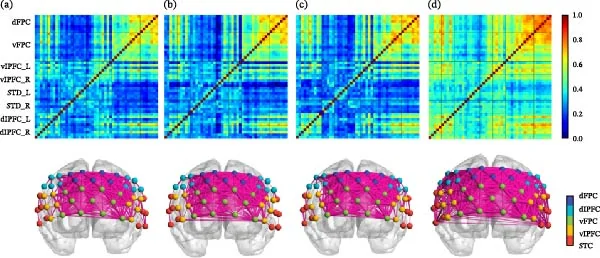
Average functional connectivity matrices (top row) and corresponding brain network graphs (bottom row) for the four groups: (a) SCZ group, (b) BD group, (c) MDD group, and (d) HC group. The functional connectivity matrices show the mean connectivity across subjects within each group, with channels arranged according to regions of interest (ROIs). The brain network graphs are constructed from thresholded connectivity matrices (threshold = 0.4), where nodes represent fNIRS channels and edges indicate functional connections stronger than 0.4. Node colors correspond to different brain regions.

FC differences across the four groups were evaluated using statistical comparisons with FDR correction to control for multiple comparison errors (Table 7). The analysis revealed a distinct pattern where all three psychiatric disorder groups (BD, D, and SP) exhibited significantly reduced FC compared to HCs, particularly in links between prefrontal and temporal regions. For instance, compared to HCs, the BD group showed significantly weaker connectivity in the dlPFC_L‐STC_R pair (*t* = 9.38, *p*_FDR_ < 0.001), the D group exhibited marked decreases in the dlPFC_L‐vlPFC_L pair (*t* = 8.58, *p*_FDR_ < 0.001), and the SP group displayed significant reductions in the STC_R‐vlPFC_R pair (*t* = 9.46, *p*_FDR_ < 0.001). In contrast, direct comparisons between the psychiatric groups themselves (BD vs. D, BD vs. SP, and D vs. SP) revealed no statistically significant differences, with all relevant comparisons yielding *p*‐values greater than 0.05, suggesting a shared pattern of connectivity deficits across these disorders.

**Table 7:** Statistical comparisons of functional connectivity across healthy controls and psychiatric groups (FDR‐corrected).

ROI‐pair	**d** **f**_**between**_ = 3			Post hoc tests																							
	**d** **f**_**within**_ = 410			HC vs. BD				HC vs. MDD				HC vs. SCZ				BD vs. MDD				BD vs. SCZ				MDD vs. SP			
	**F**	**p**_**FDR**_	*η* ^2^	**t**	**p**_**FDR**_	*df*	*Cohen’s d*	**t**	**p**_**FDR**_	*df*	*Cohen’s d*	**t**	**p**_**FDR**_	*df*	*Cohen’s d*	**t**	**p**_**FDR**_	*df*	*Cohen’s d*	**t**	**p**_**FDR**_	*df*	*Cohen’s d*	**t**	**p**_**FDR**_	*df*	*Cohen’s d*
dlPFC_L‐dPFC_R	41.44	<0.001	0.23	8.63	<0.001	181.8	1.20	8.69	<0.001	202.2	1.20	8.08	<0.001	191.8	1.12	0.92	0.541	196.9	0.13	−0.28	0.896	200.3	−0.04	−1.13	0.439	202.9	−0.16
dlPFC_L‐dFPC	6.04	<0.001	0.04	2.98	0.007	163.7	0.41	3.30	0.003	186.8	0.46	2.85	0.010	173.4	0.39	0.68	0.656	197.3	0.09	−0.08	0.970	200.8	−0.01	−0.73	0.640	202.6	−0.10
dlPFC_L‐STC_L	42.79	<0.001	0.24	9.10	<0.001	193.2	1.26	8.46	<0.001	206.1	1.17	9.13	<0.001	198.9	1.27	0.10	0.963	199.2	0.01	0.38	0.874	201.4	0.05	0.25	0.907	203.1	0.03
dlPFC_L‐STC_R	47.37	<0.001	0.26	9.38	<0.001	189.3	1.30	9.13	<0.001	206.8	1.26	8.89	<0.001	180.0	1.23	0.87	0.563	193.3	0.12	−0.95	0.537	201.5	−0.13	−1.70	0.173	184.2	−0.24
dlPFC_L‐vlPFC_L	38.55	<0.001	0.22	7.63	<0.001	180.3	1.06	8.58	<0.001	198.4	1.19	7.35	<0.001	174.9	1.02	1.75	0.157	199.9	0.24	−0.51	0.780	202.6	−0.07	−2.25	0.052	195.6	−0.31
dlPFC_L‐vlPFC_R	43.50	<0.001	0.24	9.67	<0.001	195.3	1.34	8.52	<0.001	206.0	1.18	9.02	<0.001	190.9	1.25	0.13	0.961	190.4	0.02	−0.94	0.537	202.6	−0.13	−0.91	0.541	185.6	−0.13
dlPFC_L‐vFPC	20.26	<0.001	0.13	6.12	<0.001	177.0	0.85	5.82	<0.001	205.7	0.81	6.09	<0.001	195.5	0.85	0.55	0.754	186.1	0.08	0.47	0.809	194.7	0.07	−0.11	0.961	201.6	−0.02
dPFC_R‐dFPC	5.71	<0.001	0.04	2.90	0.009	168.5	0.40	3.34	0.002	192.4	0.46	2.52	0.026	175.8	0.35	0.83	0.580	196.6	0.11	−0.43	0.837	201.7	−0.06	−1.19	0.410	200.9	−0.16
dPFC_R‐STC_L	40.66	<0.001	0.23	8.10	<0.001	180.6	1.12	8.81	<0.001	200.3	1.22	7.71	<0.001	184.6	1.07	1.58	0.213	198.2	0.22	−0.34	0.876	202.5	−0.05	−1.86	0.128	200.4	−0.26
dPFC_R‐STC_R	34.52	<0.001	0.20	7.93	<0.001	184.4	1.10	7.49	<0.001	204.5	1.04	7.59	<0.001	167.5	1.05	0.33	0.877	195.5	0.05	−0.93	0.537	198.0	−0.13	−1.14	0.432	179.6	−0.16
dPFC_R‐vlPFC_L	29.09	<0.001	0.18	7.36	<0.001	170.1	1.02	7.18	<0.001	201.2	0.99	6.29	<0.001	185.9	0.87	0.75	0.629	188.3	0.10	−0.95	0.537	197.6	−0.13	−1.51	0.241	200.5	−0.21
dPFC_R‐vlPFC_R	26.54	<0.001	0.16	7.65	<0.001	178.5	1.06	6.52	<0.001	205.7	0.90	6.31	<0.001	190.3	0.88	−0.25	0.907	187.3	−0.03	−1.28	0.358	199.6	−0.18	−0.83	0.580	197.6	−0.12
dPFC_R‐vFPC	11.92	<0.001	0.08	5.11	<0.001	166.7	0.71	3.96	<0.001	203.1	0.55	4.52	<0.001	187.0	0.63	−0.64	0.682	181.8	−0.09	−0.37	0.876	194.5	−0.05	0.29	0.896	199.2	0.04
dFPC‐STC_L	10.65	<0.001	0.07	3.90	<0.001	154.9	0.54	3.56	0.001	178.5	0.49	4.43	<0.001	156.1	0.61	−0.16	0.946	196.3	−0.02	0.82	0.580	202.9	0.12	0.88	0.558	196.9	0.12
dFPC‐STC_R	14.84	<0.001	0.10	4.55	<0.001	167.4	0.63	5.00	<0.001	187.6	0.69	4.70	<0.001	155.6	0.65	0.93	0.537	199.5	0.13	0.02	0.997	200.2	0.00	−0.96	0.537	189.0	−0.13
dFPC‐vlPFC_L	8.98	<0.001	0.06	3.12	0.005	164.6	0.43	4.21	<0.001	178.8	0.58	3.32	0.003	165.8	0.46	1.64	0.195	202.9	0.23	0.29	0.896	202.9	0.04	−1.36	0.313	202.7	−0.19
dFPC‐vlPFC_R	8.09	<0.001	0.06	3.48	0.001	166.1	0.48	3.76	<0.001	187.3	0.52	3.26	0.003	163.3	0.45	0.64	0.682	198.7	0.09	−0.34	0.876	202.9	−0.05	−0.95	0.537	196.2	−0.13
dFPC‐vFPC	4.00	0.008	0.03	2.21	0.057	173.3	0.31	3.11	0.005	183.1	0.43	1.47	0.258	196.5	0.20	1.26	0.368	204.7	0.17	−0.69	0.656	190.6	−0.10	−1.77	0.153	198.7	−0.25
STC_L‐STC_R	46.19	<0.001	0.25	9.41	<0.001	185.9	1.31	8.94	<0.001	207.0	1.24	9.32	<0.001	194.2	1.30	0.96	0.537	185.6	0.13	0.29	0.896	200.8	0.04	−0.69	0.656	194.3	−0.10
STC_L‐vlPFC_L	27.37	<0.001	0.17	7.01	<0.001	182.4	0.97	6.08	<0.001	203.7	0.84	7.60	<0.001	189.2	1.06	−0.35	0.876	195.2	−0.05	0.95	0.537	201.7	0.13	1.17	0.416	200.0	0.16
STC_L‐vlPFC_R	39.44	<0.001	0.22	8.69	<0.001	186.9	1.21	8.10	<0.001	207.0	1.12	8.92	<0.001	197.6	1.24	0.69	0.656	186.7	0.09	0.78	0.613	199.1	0.11	−0.00	0.998	197.7	−0.00
STC_L‐vFPC	25.07	<0.001	0.16	6.55	<0.001	169.2	0.91	5.27	<0.001	198.9	0.73	7.28	<0.001	165.5	1.01	−0.82	0.580	190.5	−0.11	0.93	0.537	202.8	0.13	1.61	0.204	186.8	0.22
STC_R‐vlPFC_L	23.40	<0.001	0.15	6.61	<0.001	193.4	0.92	5.97	<0.001	205.1	0.83	6.86	<0.001	183.7	0.95	−0.24	0.907	201.4	−0.03	0.01	0.997	201.1	0.00	0.26	0.907	193.5	0.04
STC_R‐vlPFC_R	43.97	<0.001	0.24	9.57	<0.001	194.3	1.33	8.33	<0.001	206.9	1.15	9.46	<0.001	194.0	1.32	−0.15	0.951	193.0	−0.02	−0.11	0.961	203.0	−0.02	0.05	0.980	192.8	0.01
STC_R‐vFPC	32.00	<0.001	0.19	7.29	<0.001	173.9	1.01	6.72	<0.001	191.8	0.93	7.51	<0.001	161.2	1.04	−0.22	0.920	200.7	−0.03	−0.04	0.989	200.1	−0.00	0.20	0.929	190.5	0.03
vlPFC_L‐vlPFC_R	21.43	<0.001	0.14	6.26	<0.001	187.5	0.87	5.55	<0.001	205.1	0.77	6.67	<0.001	181.0	0.93	−0.19	0.929	196.9	−0.03	0.34	0.876	202.3	0.05	0.50	0.790	191.2	0.07
vlPFC_L‐vFPC	20.28	<0.001	0.13	5.98	<0.001	169.4	0.83	5.47	<0.001	193.6	0.76	5.79	<0.001	172.6	0.80	−0.11	0.961	196.2	−0.01	−0.19	0.930	202.7	−0.03	−0.06	0.980	198.2	−0.01
vlPFC_R‐vFPC	22.00	<0.001	0.14	6.50	<0.001	184.7	0.90	5.39	<0.001	193.3	0.75	6.23	<0.001	181.4	0.87	−1.12	0.440	204.9	−0.15	−0.41	0.847	202.9	−0.06	0.74	0.634	203.1	0.10

## 4. Discussion

It is noteworthy that this study demonstrates the role of interdisciplinary collaboration between medicine and engineering in exploring neuroimaging research for psychiatric disorders. In practice, in addition to psychiatrists, medical imaging diagnosticians, and technicians, more data analysts need to be involved to jointly conduct data mining. Future research will yield more neuroimaging findings that may support or even reshape the diagnostic frameworks for psychiatric disorders. On the other hand, verbal fluency—one of the simplest cognitive tests—compared to complex and less intuitive psychological experimental paradigms is easy to administer in clinical practice and holds greater potential for widespread adoption. Combined with fNIRS, it could very likely serve as a general screening tool for patients with various psychiatric disorders. In the future, it may become a routine examination in psychiatric specialties, aiding in the auxiliary diagnosis and treatment monitoring of mental disorders. Therefore, this study provides practical evidence supporting the clinical application of fNIRS combined with verbal fluency testing in the assessment of psychiatric disorders.

The diagnostic performance achieved by integrating spatial topological features suggests that these metrics capture essential pathophysiological information that temporal features alone may miss. While the current study focuses on the collective discriminative power of the MSNF framework, the efficacy of these topological descriptors underscores their value as important tools for understanding and identifying psychiatric disorders [39, 40]. Literature indicates that psychiatric disorders are often characterized by subtle reorganizations in network topology. For instance, local efficiency is theoretically linked to the fault tolerance of information transfer, which has been reported to be altered in the emotional regulation circuits of patients with MDD [41]. Similarly, modularity indexes the balance between functional segregation and integration; disruptions in this balance [42, 43] are frequently observed in SCZ. By encoding these complex network properties, the MSNF framework potentially provides a more holistic representation of the “disconnection” syndromes [44] underlying these disorders, thereby facilitating more accurate cross‐disease classification.

The present results need to be interpreted with caution given several methodological and practical constraints that may influence both performance estimation and translational relevance. The study was conducted within a single clinical center, and site‐specific recruitment characteristics, demographic composition, and diagnostic routines may have shaped the observed fNIRS signal patterns. Consequently, the extent to which these findings generalize to other clinical environments or populations remains uncertain and requires independent replication. Specifically, the absence of women in the HC group is a limitation that may restrict generalizability and potentially confound sex effects; however, our supporting male‐only sensitivity analyses (Section S7) suggest that this demographic restriction does not fundamentally alter the main diagnostic classification conclusions of the present work. On the other hand, at the beginning of this study, we hoped to collect more samples. Considering the different disease states of patients with various mental disorders and the differing convenience of collecting patients from outpatient and inpatient departments, we have made maximum concessions in the assessment of various symptom scales, hoping to obtain certain results through near‐infrared data analysis. In the later stage, we will continue to improve based on the preliminary work of this project. The scale assessments will be strictly limited to observer‐rated scales, which may extend the overall research time, with the aim of obtaining thousands of samples to continue verifying the clinical diagnostic significance of near‐infrared for mental disorders.

An additional limitation concerns the discordance between the clinician‐assigned MDD labels and manic symptom ratings in 13 of 105 patients with MDD (12.4%), indicating that a nontrivial proportion of the MDD labels may be diagnostically uncertain. Supporting outer‐fold predictions classified nine cases as MDD and four as BD, while exclusion‐based analyses yielded MDD‐versus‐BD and four‐class performance comparable to the corresponding full‐sample results (Tables S5–S7). Although these findings suggest that the overall results were not substantially driven by this subgroup, they do not resolve the uncertainty of the reference labels. Consequently, the reported MDD‐versus‐BD performance should be interpreted cautiously because agreement with the assigned MDD labels may partly reflect the clinical labeling convention, whereas predictions closer to BD are counted as errors under the current reference standard. Model outputs should therefore be interpreted as similarities to learned fNIRS patterns rather than as substitutes for longitudinal clinical diagnosis.

Variability related to clinical status was not explicitly accounted for in the current analysis. Participants differed in medication exposure, illness duration, and symptom state, factors that are known to modulate cerebral hemodynamics and FC. This issue is particularly relevant for BD, where neural responses can vary substantially across disease phases. Without stratification or longitudinal sampling, it is difficult to disentangle stable disorder–related features from state‐ or treatment‐dependent effects, which may have contributed to the overlap observed between certain diagnostic groups.

Interpretation is further constrained by technical characteristics of fNIRS. The technique is sensitive to superficial blood flow and systemic physiological fluctuations, and its limited penetration depth restricts measurements to cortical regions. Although standard preprocessing procedures were applied, complete separation of cortical and extracerebral components remains challenging. In addition, the verbal fluency task involves overt speech, making the recordings vulnerable to motion‐related artifacts arising from head movement, facial muscle activity, and probe displacement. Residual motion effects may, therefore, have influenced both feature extraction and FC estimation.

From a validation standpoint, the absence of an independent external cohort and test–retest assessment limits conclusions regarding robustness and reliability. While NCV provides an internal estimate of generalization, it does not establish stability across time or across clinical settings. Whether the extracted multiscale features remain consistent under repeated measurements or perform similarly in unseen samples remains an open question.

These considerations have direct implications for clinical translation. At the current stage, the proposed framework should be viewed as an exploratory and supportive analytic approach rather than a clinically deployable diagnostic instrument. Progress toward application will require replication in independent datasets, multicenter validation to assess site robustness, and prospective clinical studies designed to evaluate performance under real‐world conditions. Longitudinal and test–retest designs will be essential to determine reliability and sensitivity to clinically meaningful changes. Until such evidence is available, claims regarding diagnostic utility should remain appropriately cautious.

## 5. Conclusion

This study proposes a MSNF framework integrating temporal dynamics and graph‐based topological features from fNIRS signals during verbal fluency tasks to enhance the classification of major psychiatric disorders. Empirical evaluation across binary, ternary, and quaternary classification tasks demonstrates that MSNF significantly improves diagnostic accuracy, particularly in differentiating depression and SCZ from HCs. Second‐order graph features and regional temporal descriptors offer complementary discriminative power, strengthening model robustness and interpretability. FC analyses reveal disease‐specific disruptions in prefrontal‐temporal circuits, reinforcing the neurobiological relevance of the extracted features. Notably, classifying BD and SCZ remains challenging due to shared connectivity alterations, with the underlying causes attributing to both neurobiological overlap and methodological limitations. Specifically, neurobiologically, BD and SCZ share genetic vulnerabilities and exhibit similar dysfunctions in DMN connectivity, which are factors that blur the neurobiological and classification boundaries between the two disorders. Methodologically, to mitigate this limitation, future studies could incorporate multimodal imaging techniques (e.g., combining fNIRS with EEG) or adopt longitudinal monitoring designs; such approaches may better capture the distinct temporal instability of mood in BD, a core characteristic that might not be fully elicited by the single‐session verbal fluency task employed in the current study. To further refine this differentiation, we plan to expand our cohort, explicitly annotating specific bipolar mood states and psychotic features. By integrating fine‐grained symptom analysis, large‐scale data, and algorithmic updates, we aim to achieve a more robust and precise separation between SCZ and BD. Despite this constraint, the proposed approach establishes a scalable and interpretable computational pipeline for psychiatric screening and cross‐diagnostic investigation.

Finally, we also raise a clinically meaningful question for future studies to explore and address: Can fNIRS become a routine clinical assessment in the future, aiding in the diagnosis of various psychiatric disorders while also monitoring both treatment effects and corresponding changes in brain function? Future research is needed to empirically answer this clinical question.

## Funding

This work was supported by the Sichuan Province Civil Affairs Department Project (Grant scmzflc 2022‐1); the STI2030‐Major Projects (Grant 2021ZD0200600); the 2022 Sichuan University‐Dazhou Municipal People’s Government School‐City Strategic Cooperation Special Fund Project, Scientific and Technological Innovation R&D Project (Grant 2022CDDZ‐22); and 1·3·5 project for disciplines of excellence–Clinical Research Incubation Project, West China Hospital, Sichuan University (Grant 2022HXFH029).

## Conflicts of Interest

The authors declare no conflicts of interest.

## Supporting Information

Additional supporting information can be found online in the Supporting Information section.

## Supporting information


**Supporting Information** Supporting Information is provided in a separate file and includes additional details on participant selection, fNIRS preprocessing and quality control, the MSNF feature–extraction algorithm, permutation testing, covariate sensitivity analyses, male‐only subgroup analysis, diagnostic‐label sensitivity analysis, and the software environment. It contains Figures S1–S8, Tables S1–S7, and Supporting Algorithm 1.

## Data Availability

The data that support the findings of this study are available upon request from the corresponding author. The data are not publicly available due to privacy or ethical restrictions.
