# Supplementary material for: Cross‐Disease Classification of Depression, Bipolar Disorder, and Schizophrenia Using Multiscale Spatiotemporal Network Fusion Features From fNIRS
Source: Depress Anxiety. 2026 Oct 6;2026:9921579. doi: 10.1155/da/9921579 (PMC13639845; doi:10.1155/da/9921579)
Supplement: Supplementary file 1 [file DA-2026-9921579-s001.pdf]

Supporting Information

Cross-Disease Classification of Depression, Bipolar Disorder, and Schizophrenia Using Multiscale Spatio-Temporal Network Fusion Feature from fNIRS

Yajing Meng[a], Yulu Yang[a], Qing Li[a], Qian Fu[b], Mingyao Gao[b], Ling He[b], Jing Zhang[b], and Changjian Qiu[b, *]

[a]*Mental Health Center, West China Hospital of Sichuan University, Chengdu 610041, China.*

[b]*College of Biomedical Engineering, Sichuan University, Chengdu 610065, China*

[*]*Corresponding author: qiuchangjian@wchscu.edu.cn*

Contents

S1 Participant Selection 2

S2 Processing Pipeline 4

S3 Detailed Quality Control Report 5

S4 Multiscale Spatio-Temporal Network Fusion Algorithm 6

S5 Permutation Test 8

S6 Sensitivity Analysis 10

S7 Subgroup Analysis: Male Patients 11

S8 Diagnostic-Label Sensitivity Analysis 17

S9 Software Environment 20

Section S1 Participant Selection

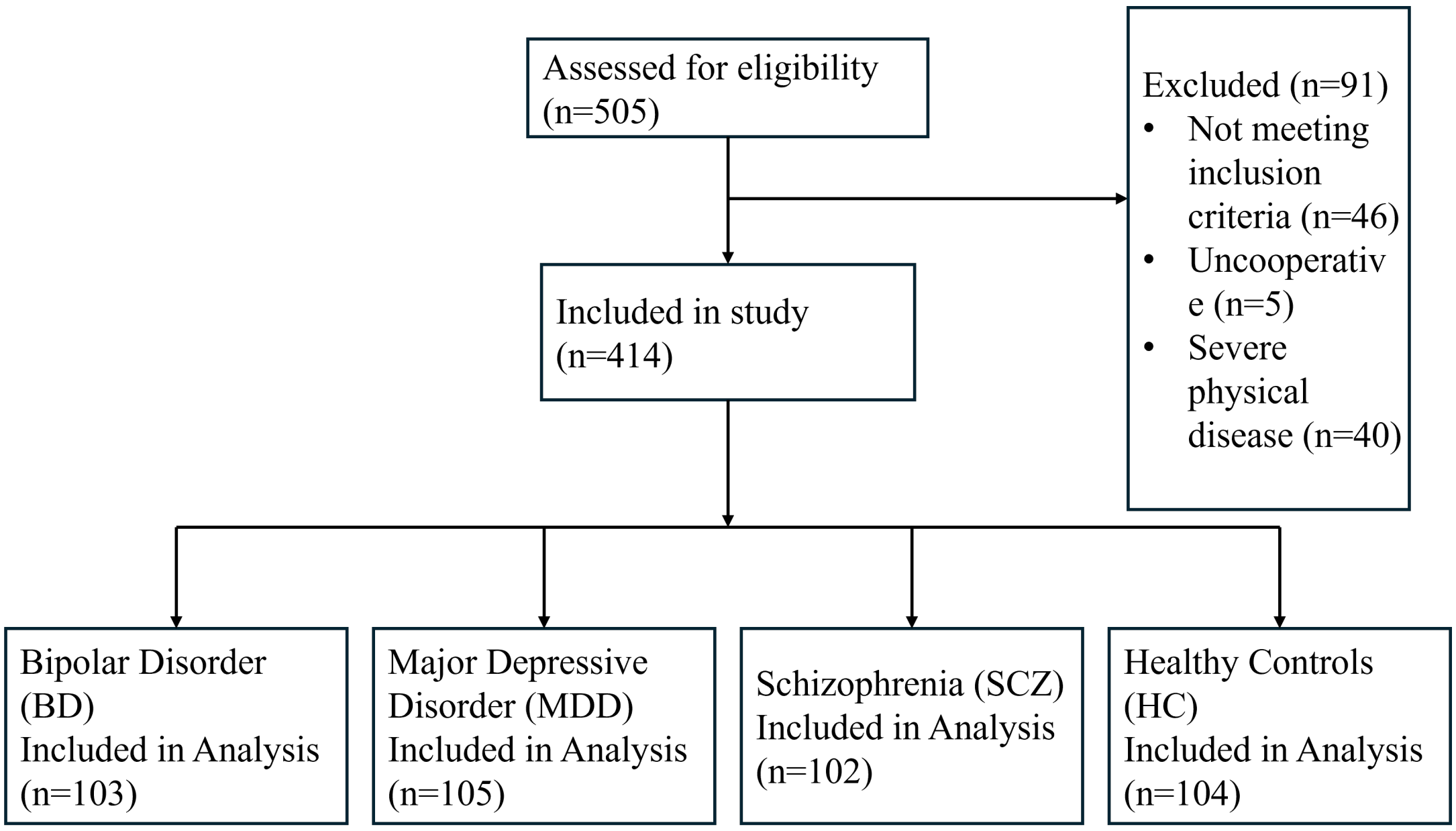

Figure S1. STROBE flow diagram depicting the participant recruitment, screening, and inclusion process.

As illustrated in the STROBE flow diagram (Figure S1), participant recruitment was conducted at the Mental Health Center, West China Hospital of Sichuan University between January 2024 and September 2024. A total of 505 candidates were initially assessed for eligibility. All patients were diagnosed by three senior psychiatrists according to DSM-5 criteria. To ensure data quality and control for comorbidities, strict exclusion criteria were applied. Out of the initial pool, 91 individuals were excluded for the following reasons: 46 did not meet specific inclusion criteria (e.g., age range of 16–65, Han Chinese ethnicity, or primary diagnostic thresholds); 40 were excluded due to severe physical diseases (e.g., history of traumatic brain injury, neurological disorders, or somatic conditions interfering with hemodynamic measurements); and 5 were excluded for being uncooperative or unable to complete the experimental tasks. The final cohort comprised 414 participants included in the statistical analysis, consisting of 103 individuals with Bipolar Disorder (BD), 105 with Major Depressive Disorder (MDD), 102 with Schizophrenia (SCZ), and 104 Healthy Controls (HC).

For clinical symptom assessment, a comprehensive battery of validated scales was employed. Depression severity was evaluated using the Hamilton Depression Rating Scale (HAMD) or

the Zung Self-Rating Depression Scale (SDS), while anxiety symptoms were assessed using the Hamilton Anxiety Rating Scale (HAMA) or the Zung Self-Rating Anxiety Scale (SAS). Manic symptoms were measured using the Young Mania Rating Scale (YMRS), and psychotic symptoms were evaluated using the Positive and Negative Syndrome Scale (PANSS) and the Brief Psychiatric Rating Scale (BPRS). Given the naturalistic outpatient setting, the specific scales administered varied based on clinical orders. To address this heterogeneity, raw scores from different instruments measuring the same construct were converted into standard scores. These standardized metrics were utilized in all subsequent analyses to ensure comparability and provide a unified measure of symptom severity across the cohort.

Section S2 Processing Pipeline

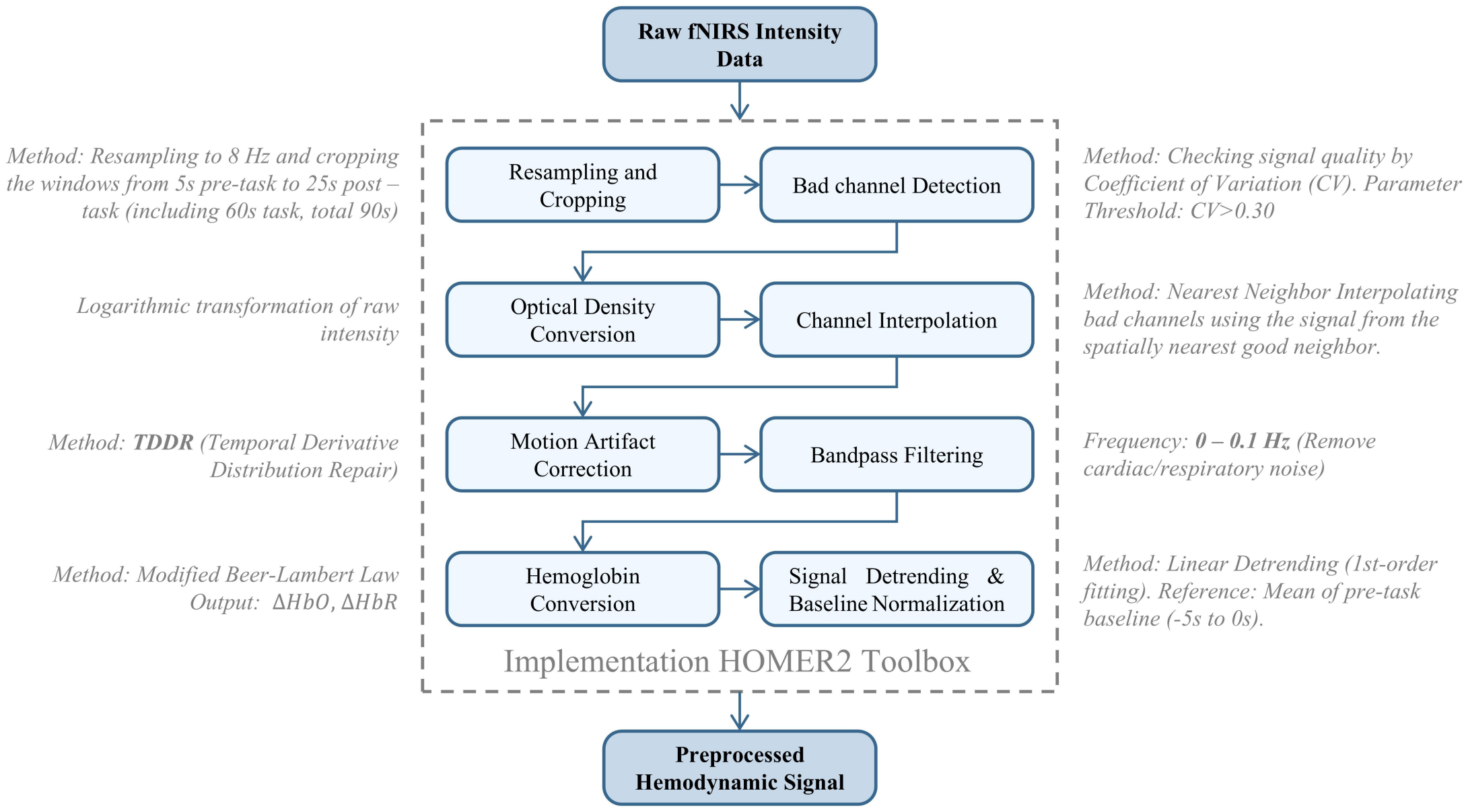

Figure S2. Flowchart of the fNIRS data preprocessing pipeline.

As illustrated in Figure S2, the fNIRS data preprocessing was conducted using the HOMER2 toolbox to ensure signal integrity and remove artifacts. The raw intensity data were first resampled to 8 Hz and cropped to include the period from 5 seconds pre-task to 25 seconds post-task (encompassing the 60-second task), resulting in a total duration of 90 seconds. To address signal quality, the Coefficient of Variation (CV) was calculated to identify artifact-contaminated channels (threshold: $CV > 0.30$), the detailed report can be found in the next section. The intensity signals were then converted into optical density through logarithmic transformation, after which the identified bad channels were interpolated using the Nearest Neighbor method based on the spatially nearest good neighbor. Crucially, the Temporal Derivative Distribution Repair (TDDR) method was applied to correct for motion artifacts. To mitigate physiological noise such as cardiac and respiratory cycles, a bandpass filter with a frequency range of 0–0.1 Hz was applied. The filtered optical density data were transformed into oxygenated (ΔHbO) and deoxygenated (ΔHbR) hemoglobin concentrations using the Modified Beer-Lambert Law. Finally, the hemodynamic signals underwent detrending via 1st-order polynomial fitting and were normalized against the baseline mean calculated from the 5-second pre-task period.

Section S3 Detailed Quality Control Report

This section presents the quality control metrics derived from the raw fNIRS light intensity signals. Given the three-wavelength configuration of the fNIRS system, quality was assessed across a total of 144 discrete channels (48 spatial ROIs × 3 wavelengths). Channel signal integrity was evaluated using the Coefficient of Variation (CV), with a threshold of $CV > 0.30$ indicating artifact contamination. The following tables detail the distribution of excluded channels across the total population ($N = 414$) and diagnostic groups prior to interpolation.

Table S1. Data Quality Overview and Distribution. (A) Summary of global quality metrics. (B) Number of subjects categorized by count of bad intensity channels.

**(A) Quality Summary**		**(B) Bad Channel Distribution**					
Metric	Value	Range	0	1–5	6–10	11–20	> 20
Total Subjects	414	Count	359	39	10	6	0
Bad Ch Rate	0.43%						
Mean Bad/Subj	0.62±2.3	*Note: 86.7% of subjects had zero bad channels.*					

Table S2. Frequent Bad Channels and Group Comparison. (A) Top 10 channels with highest failure rates. (B) Comparison of bad intensity channel counts across diagnostic groups.

**(A) Top Frequent Bad Channels**			**(B) Group Quality Comparison**				
Channel	Count	%	Group	N	Mean	SD	Max
Ch 105	11	2.7%	BD	103	0.50	2.28	19
Ch 57	9	2.2%	MDD	105	0.48	1.46	9
Ch 2, 9, 98	7–8	~1.8%	HC	104	0.94	2.99	19
Ch 20, 50, 58	6	1.5%	SCZ	102	0.57	2.06	16
Ch 7, 106	5–6	~1.3%	*Kruskal-Wallis Test:* $H = 2.39$, $p = 0.496$ *(NS)*				

Section S4 Multiscale Spatio-Temporal Network Fusion Algorithm

Algorithm Algorithm 1 presents the detailed pseudocode for the Multiscale Spatio-Temporal Network Fusion (MSNF) feature extraction framework. The procedure systematically integrates information from two complementary dimensions: temporal local dynamics and spatial topological structures. First, processed fNIRS signals are analyzed channel-wise to extract statistical, morphological, and higher-order temporal features that characterize local hemodynamic responses. Second, to model spatial connectivity, functional brain networks are constructed using Spearman correlation, and global and nodal graph metrics are computed across a range of sparsity thresholds (multiscale approach) to obtain robust topological features. These diverse feature sets are concatenated to form a final high-dimensional representation vector for each subject, providing a comprehensive basis for cross-disease classification.

Algorithm 1 Multiscale Spatio-Temporal Network Fusion (MSNF) Feature Extraction

Require: Preprocessed fNIRS data $\mathcal{D}$; Subject set $\mathcal{S}$; Channel set C ($N_{ch} = 48$); Time points T; Sparsity thresholds $\mathcal{T} = \{0.2, \ldots, 0.5\}$;
Ensure: Fused feature matrix $\mathbf{X}_{\text{MSNF}} \in \mathbb{R}^{|\mathcal{S}| \times D_{\text{feat}}}$
Initialization: Feature set $\mathcal{L} \leftarrow \emptyset$
for each subject $s \in \mathcal{S}$ **do**
  Load full signal $\mathbf{X}_{\text{full}} \in \mathbb{R}^{T \times N_{ch}}$ from $\mathcal{D}$ for subject s
  Define temporal window: $\mathbf{X}_{\text{win}} \leftarrow \mathbf{X}_{\text{full}}[5\text{s} : 65\text{s}, :]$ ▷ 60s task-locked window
  Initialize subject feature vector: $\mathbf{v}_s \leftarrow \emptyset$
  // Part 1: Temporal Local Hemoglobin Dynamic (Paper Sec 2.3.2)
  for each channel $i \in C$ **do**
    Extract channel signal $x_i(t)$ from $\mathbf{X}_{\text{win}}$
    Basic Params: μ (Mean), Slope, I (Integral), C (Centroid)
    Morphology: $peak$, $valley$, WF (Waveform Factor)
    Higher-Order: σ^2 (Variance), $Skew$ (Skewness), $Kurt$ (Kurtosis)
    $\mathbf{v}_{\text{temp}}^{(i)} \leftarrow [\mu, \text{Slope}, I, C, peak, valley, WF, \sigma^2, Skew, Kurt]$
    $\mathbf{v}_s \leftarrow \mathbf{v}_s \cup \mathbf{v}_{\text{temp}}^{(i)}$ ▷ Concatenate channel features
  end for
  // Part 2: Spatial Hierarchy Connectivity Pattern (Paper Sec 2.3.2)
  Compute Functional Connectivity (FC) matrix $\mathbf{R}$ from $\mathbf{X}_{\text{full}}$ using Spearman correlation:
    $A_{i,j} = 1 - \frac{6 \sum d_i^2}{n(n^2-1)}$ ▷ Eq. in Paper Sec 2.3.2
  Initialize graph feature accumulator: $\mathbf{G}_{\text{list}} \leftarrow \emptyset$
  for each threshold $\tau \in \mathcal{T}$ **do**
    Binarize adjacency matrix: $\mathbf{A}_\tau \leftarrow \mathbb{I}(|\mathbf{R}| \geq \tau)$
    $\mathbf{g}_\tau \leftarrow \textsc{CompGraphMetrics}(\mathbf{A}_\tau)$
    $\mathbf{G}_{\text{list}} \leftarrow \mathbf{G}_{\text{list}} \cup \mathbf{g}_\tau$
  end for
  $\mathbf{v}_{\text{spatial}} \leftarrow \text{Mean}(\mathbf{G}_{\text{list}})$ ▷ Average across multiscale thresholds
  $\mathbf{v}_s \leftarrow \mathbf{v}_s \cup \mathbf{v}_{\text{spatial}}$
  $\mathcal{L} \leftarrow \mathcal{L} \cup \{\mathbf{v}_s\}$
end for
$\mathbf{X}_{\text{MSNF}} \leftarrow \textsc{Stack}(\mathcal{L})$
return $\mathbf{X}_{\text{MSNF}}$

procedure $\textsc{CompGraphMetrics}(\mathbf{A})$ ▷ See Table 2 in Paper
  Node Profile: Degree (k_i), Betweenness Centrality (BC_i)
  Local Org: Clustering Coeff (C_i), Local Efficiency ($E_{\text{loc},i}$)
  Global Integ: Global Efficiency (E_{glob}), Modularity (Q)
  return Concatenation of all metrics averaged or flattened
end procedure

Section S5 Permutation Test

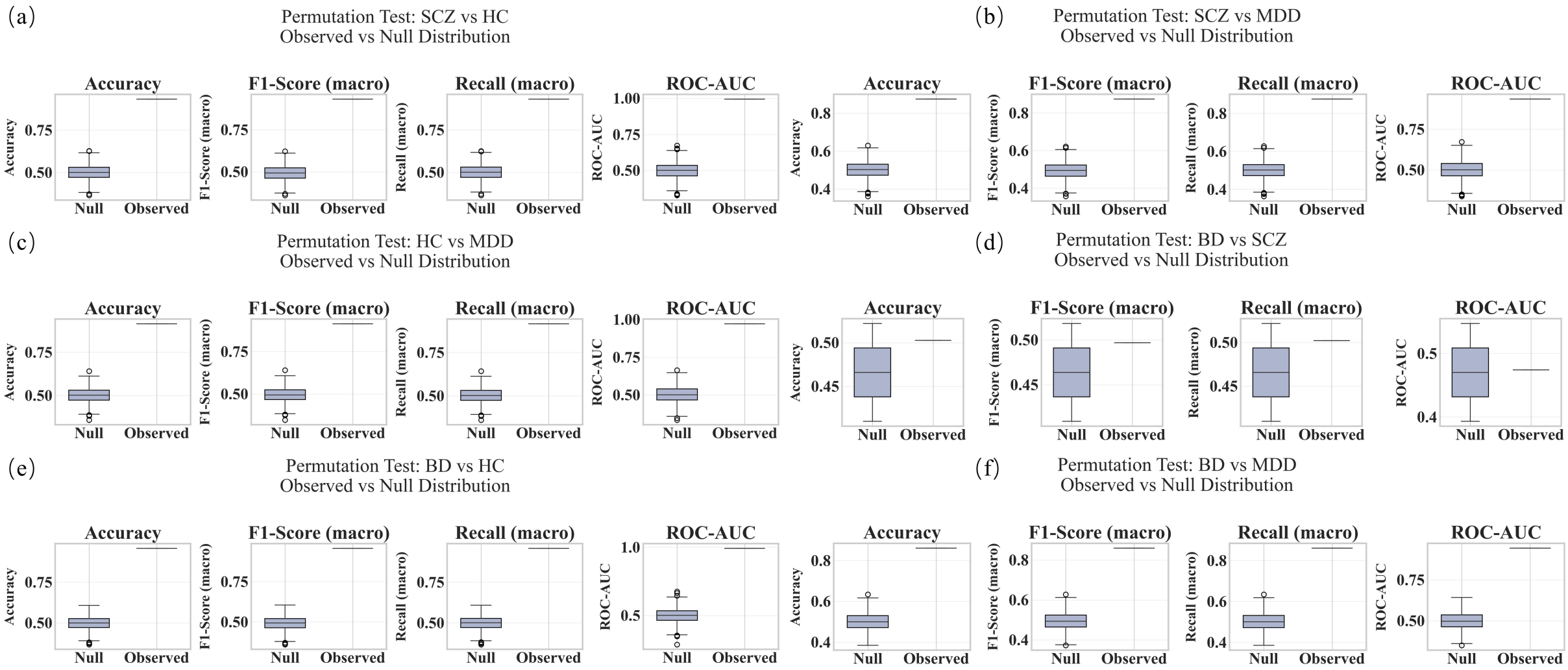

Figure S3. Observed vs. null performance distributions for Random Forest binary classification. Blue boxes represent the null distribution generated from 1000 permutations; the black line indicates the actual model performance. The Random Forest model demonstrates performance significantly distinguishable from chance in 5 out of 6 tasks (a-c, e-f), while BD vs. SCZ (d) shows overlapping distributions.

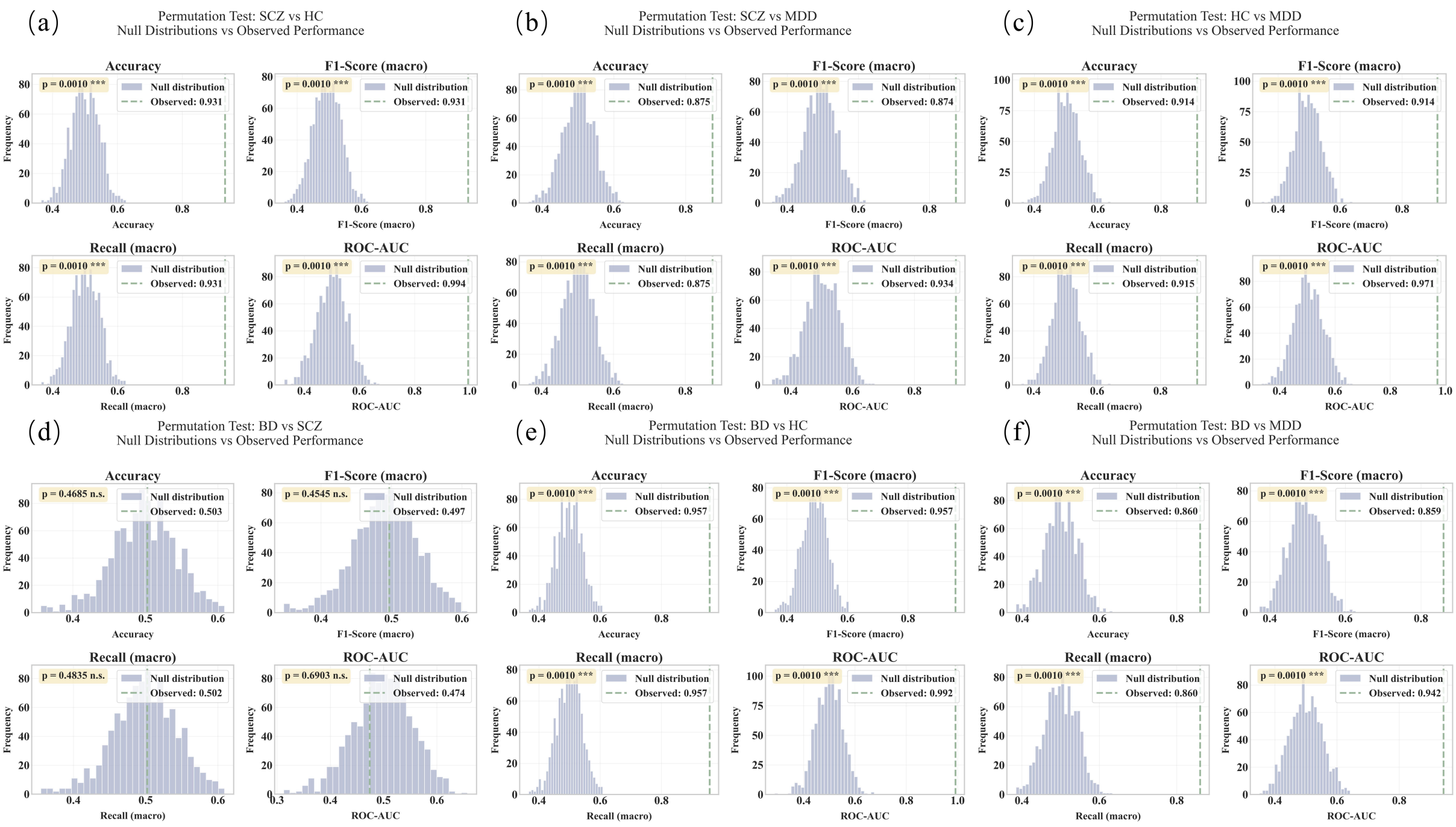

Figure S4. Permutation test results (n = 1000) for Random Forest binary classification. Blue histograms show the empirical null distributions of performance metrics; distinct green dashed lines mark the observed performance. P-values confirm significant pathological discrimination ($p < 0.001$) for all comparisons involving HC and between SCZ vs. MDD.

To rigorously validate the reliability of our classification results and mitigate the risk that the high accuracy was due to chance or overfitting, we performed a non-parametric permutation test with 1,000 iterations for each binary classification task. In this procedure, the subject labels were randomly shuffled while maintaining the original feature matrix structure, and the Random Forest classifier was retrained and evaluated on each permuted dataset using the exact same cross-validation protocol. This generated an empirical null distribution representing the classifier's performance under the hypothesis of no association between the fNIRS features and the diagnostic groups. We then quantified the statistical significance by comparing the observed performance metrics against this null distribution.

Figures S3 and S4 visualize these comparisons across all six binary tasks. As shown in Figure S3, the boxplots reveal a clear separation between the observed performance (black line) and the null distributions (blue boxes) for most comparisons. Specifically, for tasks distinguishing patients from HC—including SCZ vs. HC, BD vs. HC, and MDD vs. HC—the observed accuracy, F1-score, and ROC-AUC consistently lie far above the upper whisker of the null distribution boxes. This separation is quantitatively confirmed in Figure S4, where the histograms illustrate that the observed performance falls completely outside the null distribution for these tasks, yielding statistically significant p-values ($p < 0.001$). Similarly, the SCZ vs. MDD task demonstrates high statistical significance, suggesting distinct hemodynamic patterns between these two conditions.

Section S6 Sensitivity Analysis

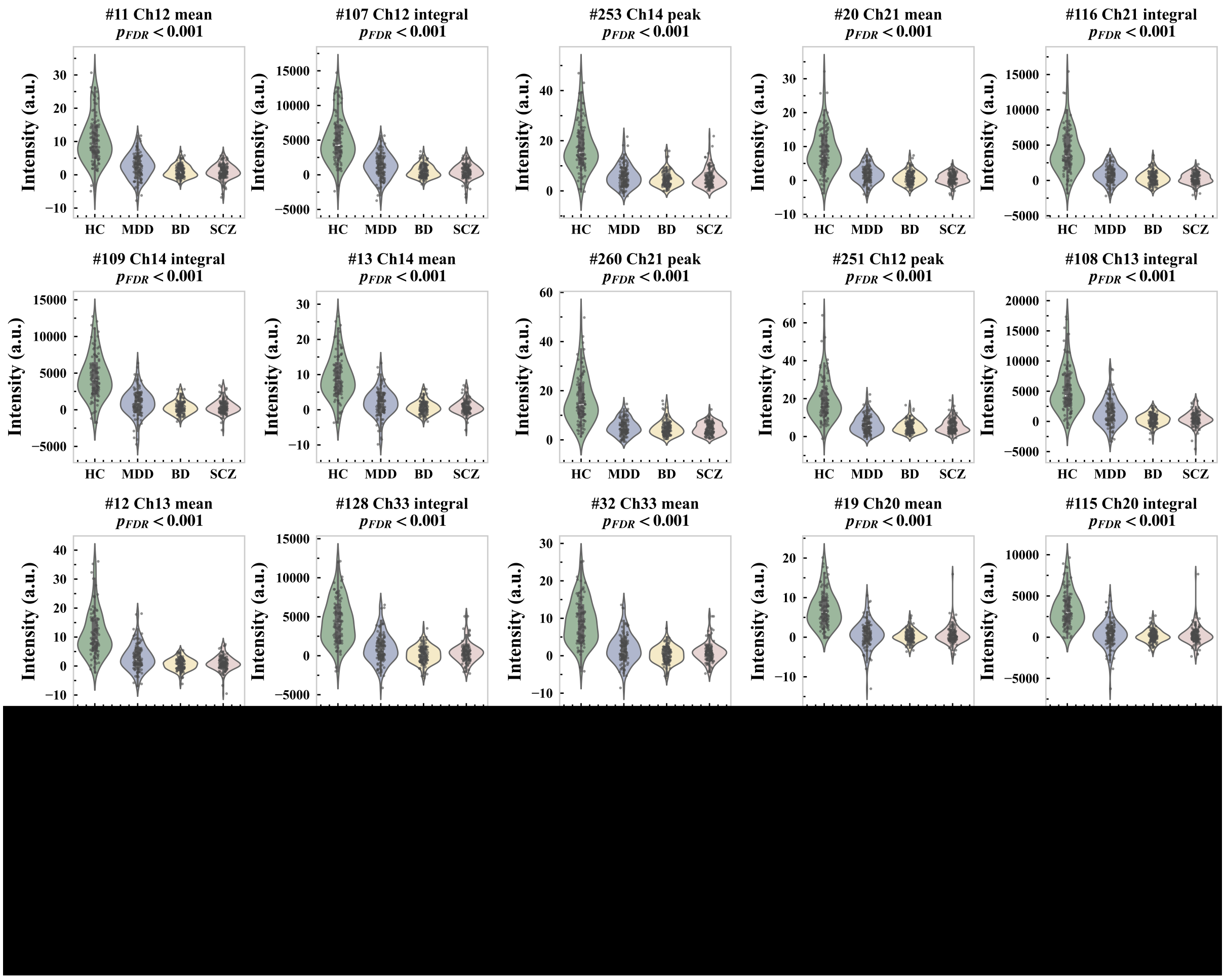

Figure S5. Top 20 discriminatory features across diagnostic groups. Violin plots with overlaid data points illustrate the distributions of significant features identified by ANCOVA ($p_{FDR} < 0.05$) after controlling for age, gender. FDR-corrected p-values are displayed above each feature. Groups: Healthy Controls (HC), Major Depressive Disorder (MDD), Bipolar Disorder (BD), Schizophrenia (SCZ).

To identify features with significant group differences while controlling for confounding factors, we performed Analysis of Covariance (ANCOVA) for each feature using the diagnostic group as the fixed factor and age and gender as covariates. The p-values for the group main effect were adjusted for multiple comparisons using the Benjamini-Hochberg False Discovery Rate (FDR) method. Features with an adjusted $p_{FDR} < 0.05$ were considered statistically significant. The distributions of the top 20 most significant features were visualized using violin plots as shown in Figure S5, overlaid with individual data points to show both the probability density and raw data distribution across the four groups (HC, MDD, BD, SCZ).

Section S7 Subgroup Analysis: Male Patients

To evaluate the robustness of our MSNF feature extraction and classification framework, we conducted a subgroup analysis restricted exclusively to male participants. This analysis aims to verify whether the observed diagnostic patterns and model performance remain consistent when gender-related variance is eliminated via cohort restriction. We evaluated the classification performance across six binary tasks under three different confounding control scenarios: (1) No Adjustment, (2) Linear Adjustment (ANCOVA-based), and (3) Support Vector Regression (SVR-based) Adjustment, the detailed results are summarized in Table S3.

Table S3. Classification Performance in the Male-Only Subgroup. Results are reported as Mean ± SD across cross-validation folds. Three confounding control scenarios are evaluated: (1) **No Adj.**, raw features without adjustment; (2) **Linear**, features adjusted using linear regression to remove covariate effects; (3) **SVR**, features adjusted using Support Vector Regression to account for potential non-linear confounding effects. Performance of SVM, Random Forest, and MLP is presented in terms of Accuracy and AUC.

Task	Scenario	Metric	SVM	Random Forest	MLP
BD vs. HC	No Adj.	Acc	0.942 ± 0.043	0.955 ± 0.059	0.910 ± 0.045
		AUC	0.988 ± 0.020	0.971 ± 0.046	0.957 ± 0.070
	Linear	Acc	0.882 ± 0.049	0.926 ± 0.088	0.859 ± 0.062
		AUC	0.930 ± 0.059	0.951 ± 0.073	0.903 ± 0.096
	SVR	Acc	0.933 ± 0.054	0.940 ± 0.082	0.896 ± 0.049
		AUC	0.984 ± 0.026	0.972 ± 0.040	0.910 ± 0.082
HC vs. SCZ	No Adj.	Acc	0.915 ± 0.073	0.955 ± 0.066	0.865 ± 0.063
		AUC	0.981 ± 0.020	0.985 ± 0.021	0.893 ± 0.063
	Linear	Acc	0.883 ± 0.076	0.903 ± 0.084	0.871 ± 0.094
		AUC	0.952 ± 0.040	0.975 ± 0.031	0.944 ± 0.061
	SVR	Acc	0.910 ± 0.059	0.909 ± 0.084	0.865 ± 0.086
		AUC	0.973 ± 0.033	0.981 ± 0.025	0.904 ± 0.086
HC vs. MDD	No Adj.	Acc	0.849 ± 0.095	0.895 ± 0.067	0.871 ± 0.069
		AUC	0.934 ± 0.057	0.971 ± 0.054	0.923 ± 0.088
	Linear	Acc	0.788 ± 0.030	0.842 ± 0.050	0.780 ± 0.023
		AUC	0.794 ± 0.124	0.929 ± 0.078	0.672 ± 0.177
	SVR	Acc	0.887 ± 0.061	0.864 ± 0.067	0.872 ± 0.089
		AUC	0.957 ± 0.038	0.909 ± 0.101	0.898 ± 1.13
SCZ vs. MDD	No Adj.	Acc	0.725 ± 0.135	0.825 ± 0.100	0.663 ± 0.138

Continued on next page

Table S3 – continued from previous page

**Task**	**Scenario**	**Metric**	**SVM**	**Random Forest**	**MLP**
		AUC	0.698 ± 0.159	0.898 ± 0.104	0.702 ± 0.124
	Linear	Acc	0.750 ± 0.097	0.813 ± 0.084	0.763 ± 0.088
		AUC	0.755 ± 0.181	0.867 ± 0.158	0.711 ± 0.160
	SVR	Acc	0.713 ± 0.126	0.800 ± 0.100	0.725 ± 0.122
		AUC	0.798 ± 0.162	0.853 ± 0.165	0.748 ± 0.160
BD vs. MDD	No Adj.	Acc	0.730 ± 0.151	0.830 ± 0.167	0.630 ± 0.159
		AUC	0.818 ± 0.155	0.879 ± 0.131	0.708 ± 0.120
	Linear	Acc	0.683 ± 0.203	0.697 ± 0.122	0.613 ± 0.180
		AUC	0.783 ± 0.176	0.851 ± 0.131	0.629 ± 0.200
	SVR	Acc	0.733 ± 0.238	0.697 ± 0.192	0.680 ± 0.085
		AUC	0.829 ± 0.178	0.885 ± 0.130	0.679 ± 0.172
BD vs. SCZ	No Adj.	Acc	0.576 ± 0.119	0.626 ± 0.029	0.554 ± 0.091
		AUC	0.567 ± 0.169	0.409 ± 0.127	0.468 ± 0.046
	Linear	Acc	0.626 ± 0.029	0.565 ± 0.115	0.604 ± 0.059
		AUC	0.636 ± 0.144	0.483 ± 0.188	0.517 ± 0.050
	SVR	Acc	0.614 ± 0.048	0.615 ± 0.032	0.601 ± 0.081
		AUC	0.608 ± 0.100	0.456 ± 0.181	0.496 ± 0.036

The discriminative power of the MSNF features for each classification task in the male subgroup is further visualized through ROC curves. As illustrated in Figure S6, the model achieves robust performance across tasks, particularly in distinguishing patients from healthy controls.

To further assess the stability of the classification results under different confounding correction scenarios, sensitivity analyses were conducted for all binary tasks. Figure S7 visualizes the AUC performance metrics across the three scenarios (No Adj., Linear, and SVR), demonstrating the robustness of our MSNF framework to various covariate adjustment methods.

Furthermore, we conducted a statistical comparison between the classification accuracies obtained in the male-only subgroup and those from the full study cohort to determine if gender-specific models significantly differ in performance. As summarized in Table S4, the differences in performance were evaluated using permutation tests. The results indicate that for the vast majority of disease discrimination tasks and classifiers, the classification accuracy in the male subgroup remains robustly consistent with the full cohort results ($p > 0.05$). A statistically

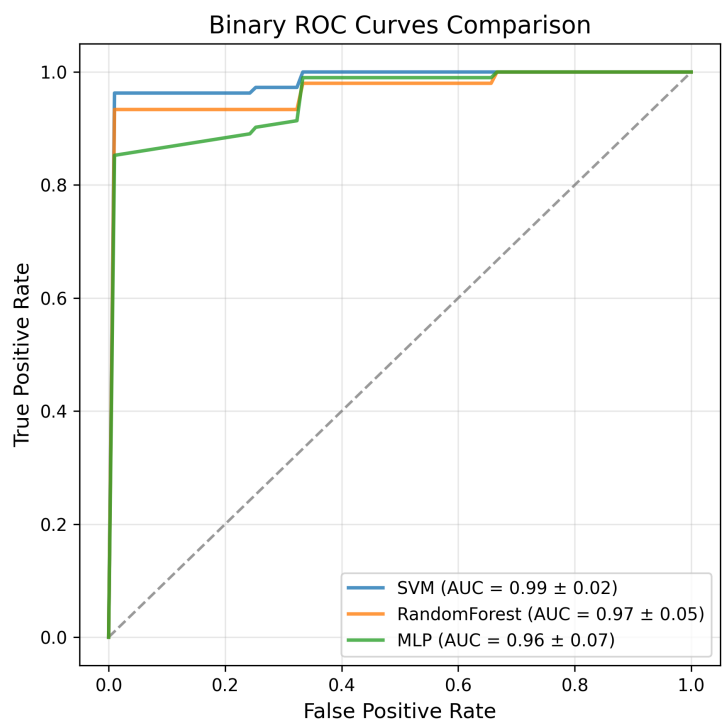

(a) BD vs. HC

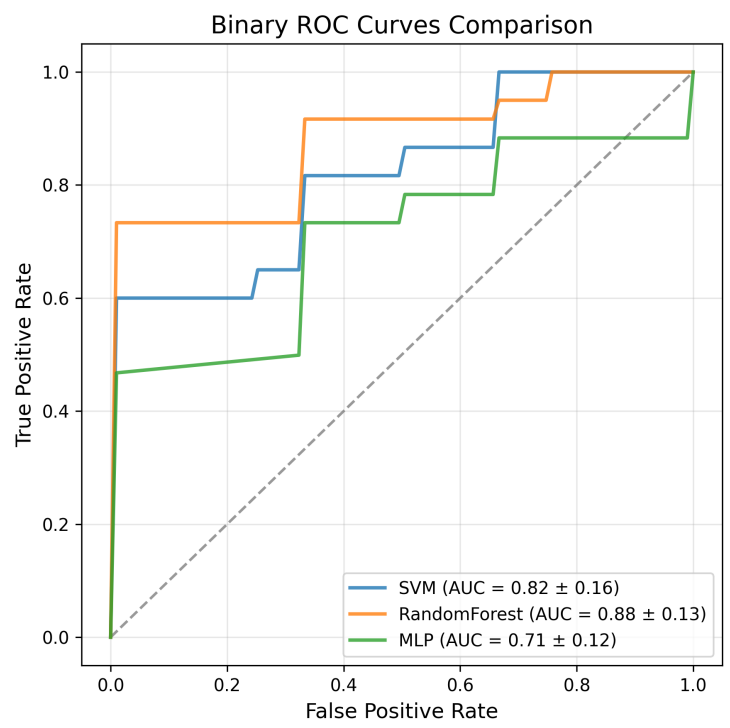

(b) BD vs. MDD

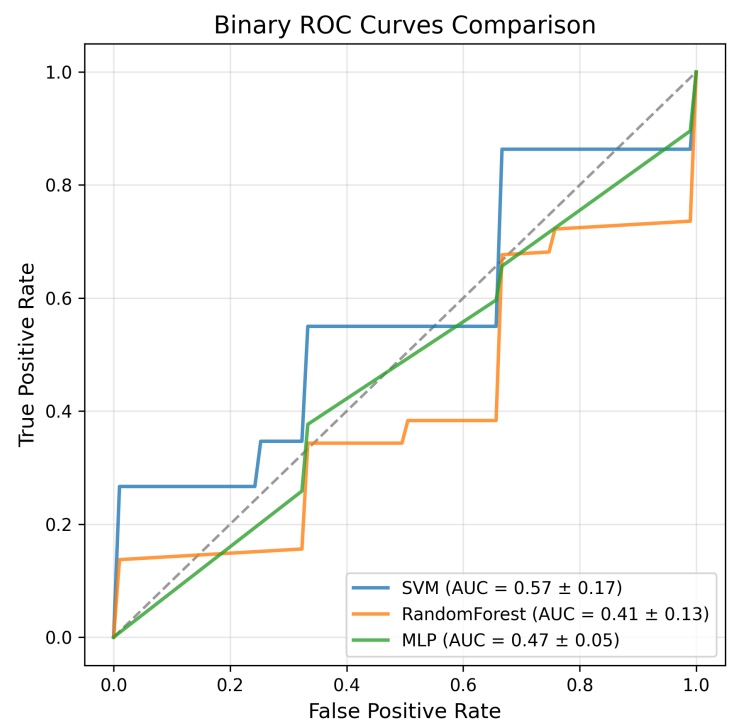

(c) BD vs. SCZ

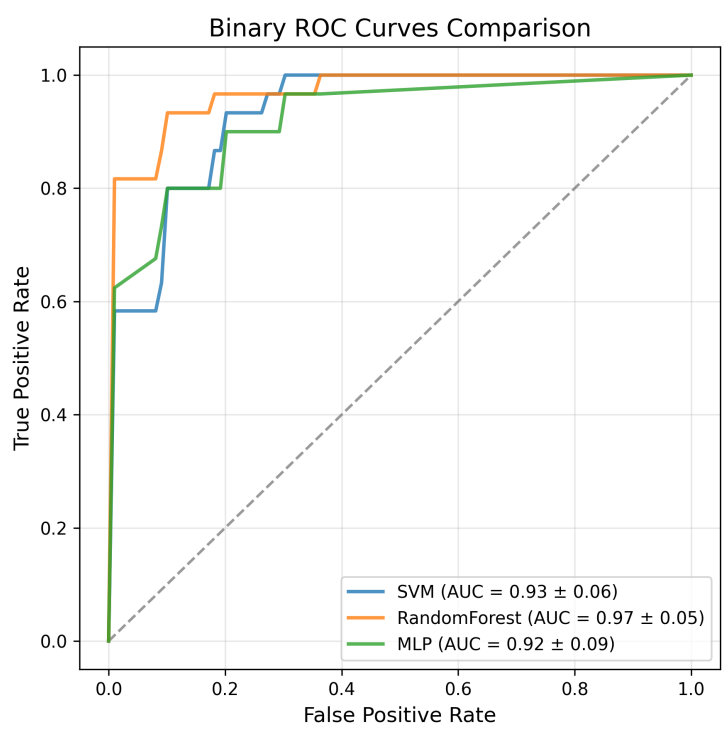

(d) HC vs. MDD

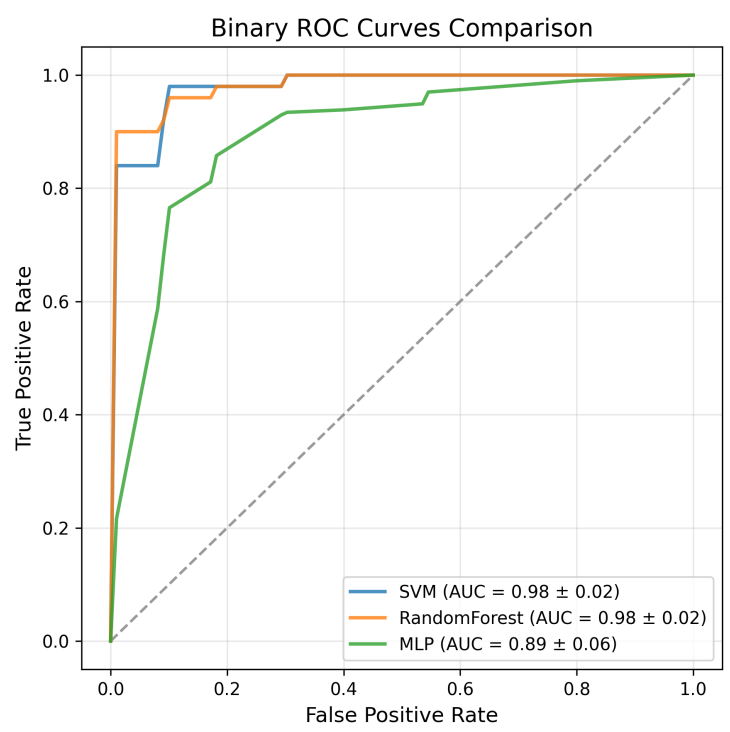

(e) HC vs. SCZ

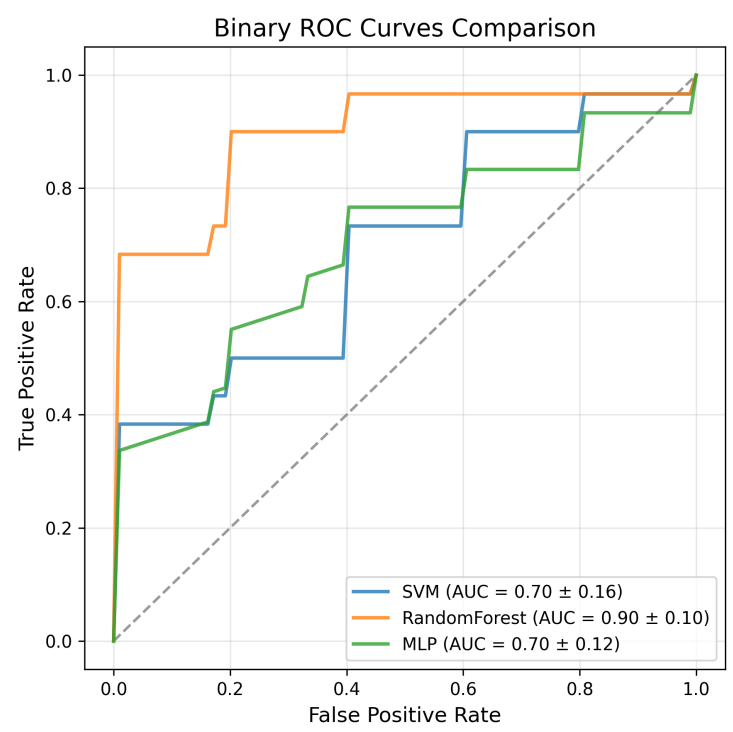

(f) SCZ vs. MDD

Figure S6. ROC curves for the cross-disease classification tasks in the male-only subgroup. Each panel represents the performance of the classification models for a specific diagnostic comparison.

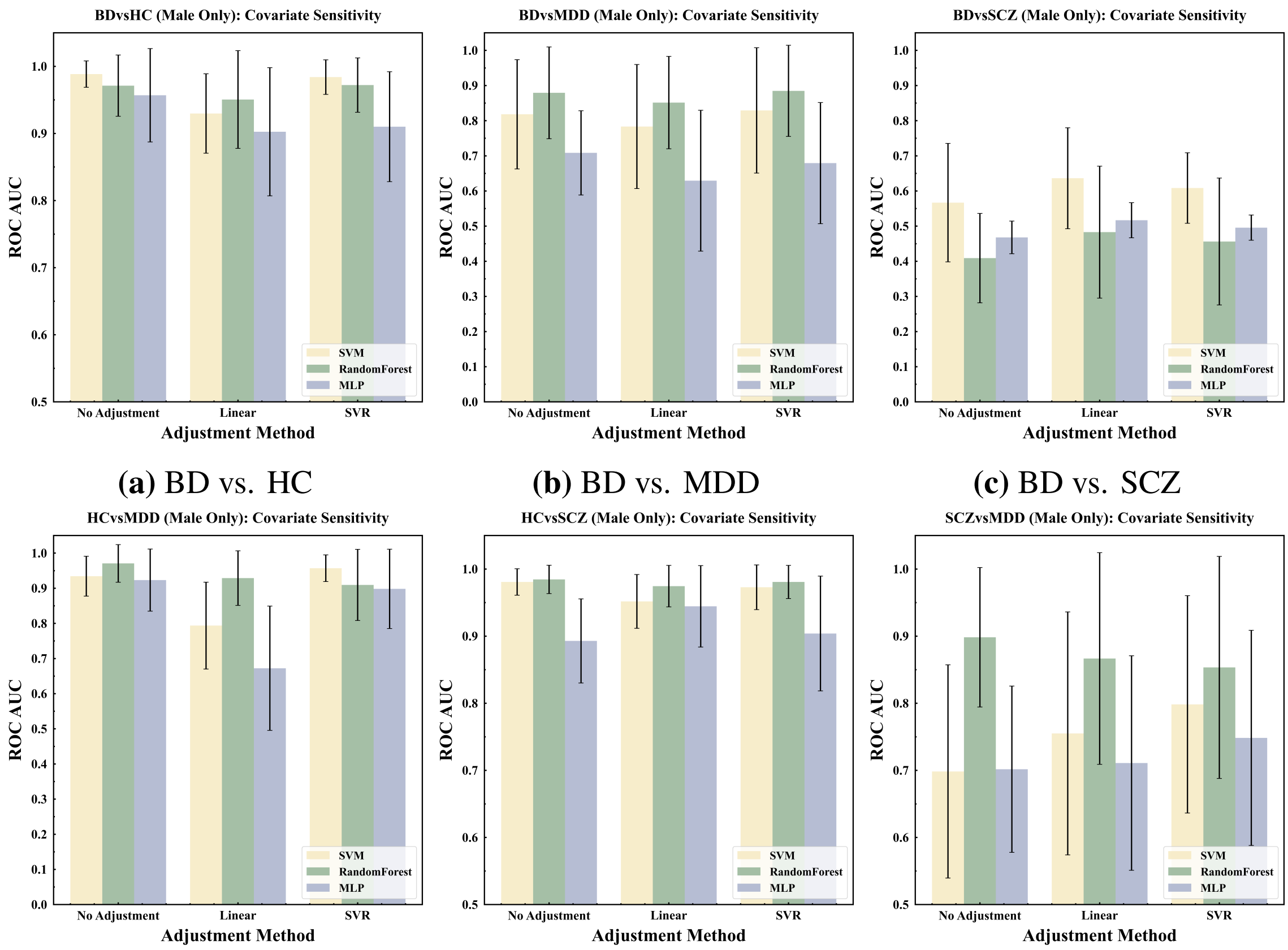

(a) BD vs. HC
(b) BD vs. MDD
(c) BD vs. SCZ
(d) HC vs. MDD
(e) HC vs. SCZ
(f) SCZ vs. MDD

Figure S7. Sensitivity analysis across different adjustment scenarios for the male subgroup. The plots compare the AUC performance across No Adjustment, Linear, and SVR-based covariate correction methods.

significant difference was observed only in the BD vs. MDD classification task using the MLP classifier (p = 0.049).

Table S4. Statistical comparison of classification accuracy between male subgroup and full cohort. This table evaluates whether restricting the analysis to male participants leads to significant performance shifts compared to the original full-sample results. Significant differences ($p < 0.05$) are marked with an asterisk (*).

Task	Classifier	Acc (Male)	Acc (Full)	Diff	p-value	Significant
BD vs. HC	MLP	0.889	0.818	+0.071	0.857	No
	RandomForest	0.941	0.945	-0.004	0.857	No
	SVM	0.919	0.843	+0.076	1.000	No
BD vs. MDD	MLP	0.641	0.780	-0.139	0.049	Yes*
	RandomForest	0.741	0.819	-0.077	0.400	No
	SVM	0.716	0.762	-0.046	0.400	No
BD vs. SCZ	MLP	0.587	0.507	+0.080	0.057	No
	RandomForest	0.602	0.559	+0.043	0.229	No
	SVM	0.606	0.508	+0.097	0.057	No
HC vs. MDD	MLP	0.841	0.731	+0.110	1.000	No
	RandomForest	0.867	0.921	-0.054	0.114	No
	SVM	0.841	0.761	+0.081	1.000	No
HC vs. SCZ	MLP	0.867	0.822	+0.044	1.000	No
	RandomForest	0.922	0.900	+0.022	0.629	No
	SVM	0.903	0.829	+0.074	1.000	No
SCZ vs. MDD	MLP	0.717	0.719	-0.002	0.857	No
	RandomForest	0.813	0.822	-0.009	0.629	No
	SVM	0.729	0.744	-0.015	0.400	No

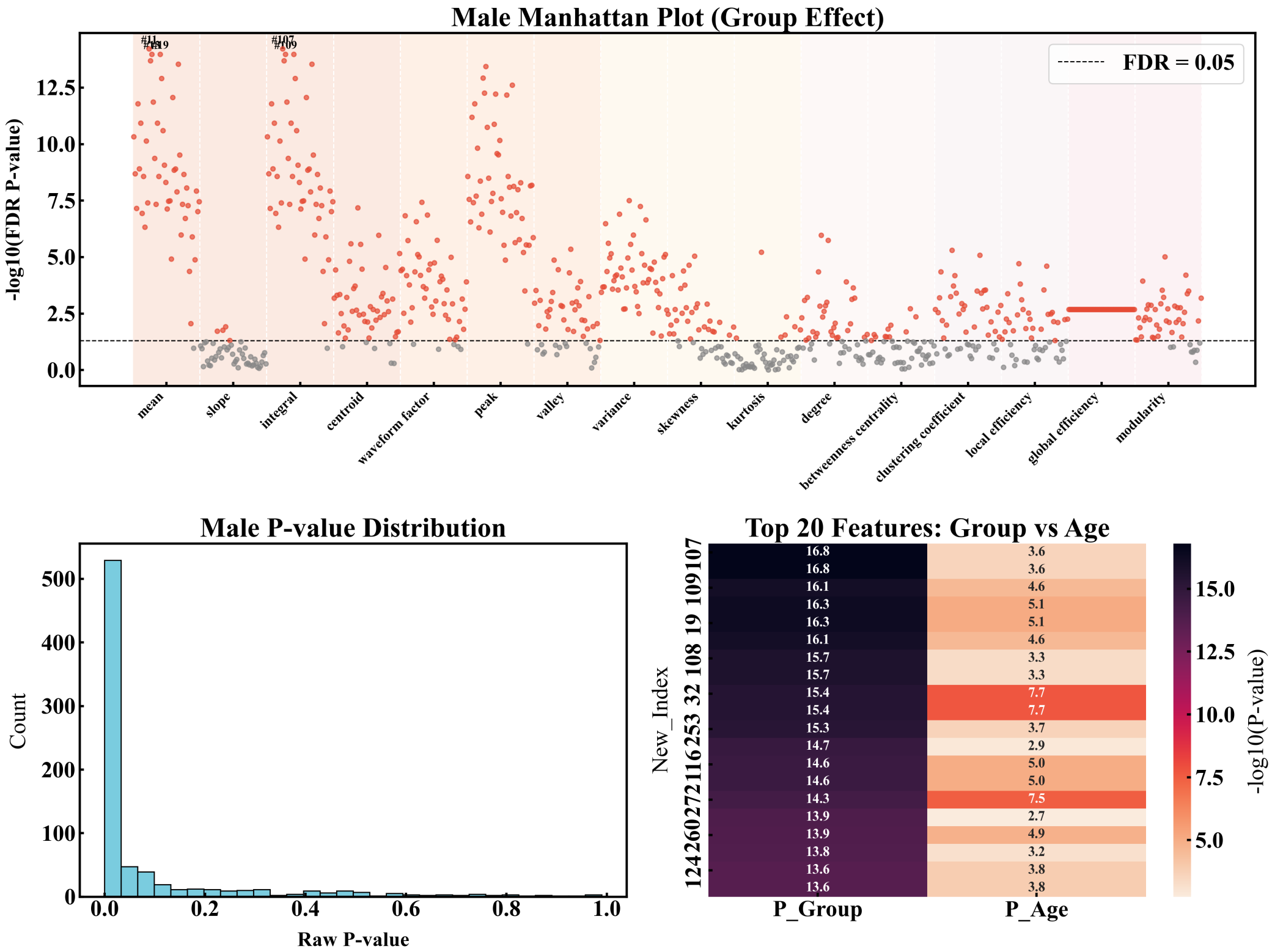

(a) Statistical summary and performance distribution.

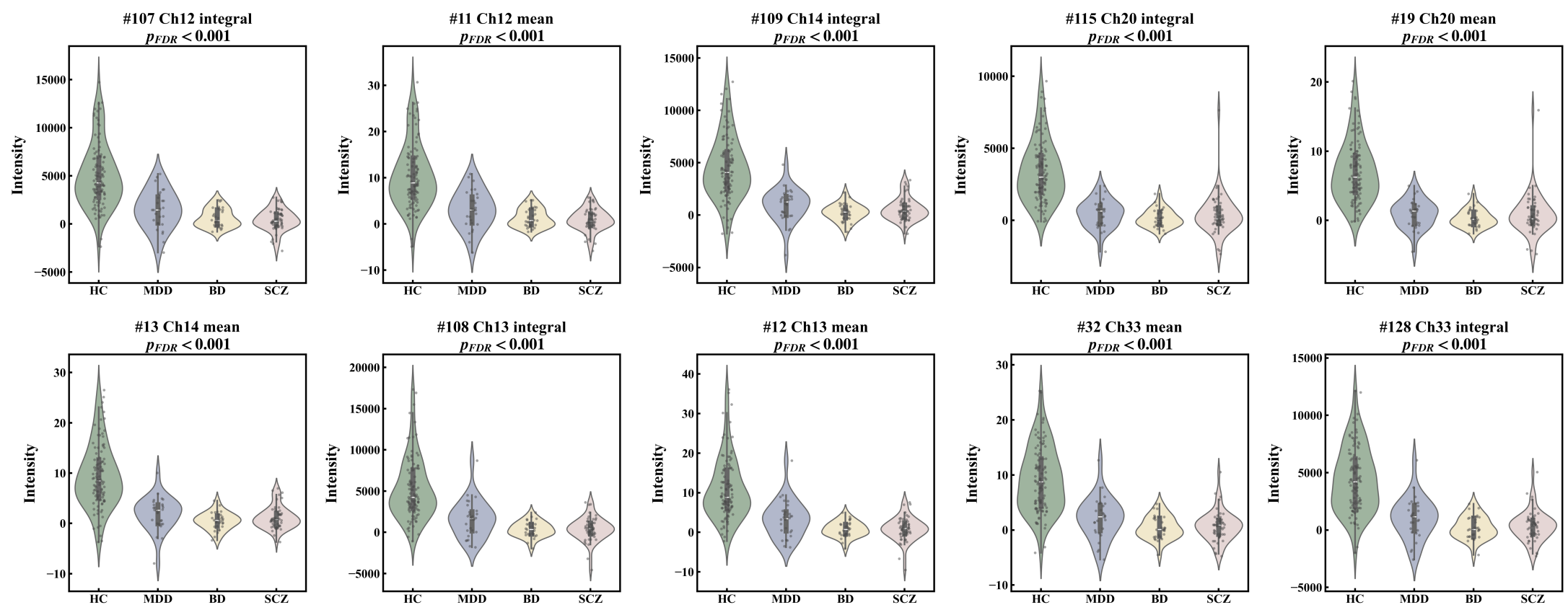

(b) Top 20 discriminatory features across diagnostic groups.

Figure S8. Summary of model performance and feature importance in the male subgroup analysis. (a) Overview of diagnostic cross-comparison accuracy; (b) Distribution patterns of the most significant MSNF features.

Section S8 Diagnostic-Label Sensitivity Analysis

To assess the potential influence of the 13 MDD cases with discordant manic symptom ratings, we examined their outer-fold predictions in a supplementary MDD-versus-BD analysis and repeated both the MDD-versus-BD binary classification and the four-class classification after excluding these cases. Individual predictions and comparisons between the full-sample and exclusion analyses are presented in Tables S5 to S7.

Table S5. Individual outer-fold predictions for the 13 MDD cases with discordant manic symptom ratings using the Random Forest model. Predictions were generated from the outer test folds of nested cross-validation, with each participant excluded from model fitting and hyperparameter selection. MDD, major depressive disorder; BD, bipolar disorder; YMRS, Young Mania Rating Scale.

Case	Clinical label	YMRS score	Outer test fold	Predicted label	MDD probability	BD probability
Case 1	MDD	32	4	BD	0.4967	0.5033
Case 2	MDD	36	10	BD	0.2997	0.7003
Case 3	MDD	40	3	MDD	0.6888	0.3112
Case 4	MDD	50	6	MDD	0.5830	0.4170
Case 5	MDD	46	7	MDD	0.6741	0.3259
Case 6	MDD	40	4	MDD	0.6333	0.3667
Case 7	MDD	35	3	BD	0.4598	0.5402
Case 8	MDD	44	1	MDD	0.6134	0.3866
Case 9	MDD	34	2	MDD	0.5640	0.4360
Case 10	MDD	54	8	MDD	0.5733	0.4267
Case 11	MDD	34	10	BD	0.4980	0.5020
Case 12	MDD	26	5	MDD	0.5300	0.4700
Case 13	MDD	52	3	MDD	0.7667	0.2333

Table S6. Comparison of Random Forest MDD-versus-BD binary classification performance between the primary full-sample analysis and the sensitivity analysis excluding 13 MDD cases with discordant manic symptom ratings. Values are reported as mean ± SD across the outer test folds of nested cross-validation. MDD, major depressive disorder; BD, bipolar disorder; AUC, area under the receiver operating characteristic curve.

Analysis	MDD	BD	Accuracy	AUC
Primary full-sample analysis	105	103	0.86 ± 0.06	0.94 ± 0.03
Exclusion sensitivity analysis	92	103	0.85 ± 0.07	0.93 ± 0.05

Table S7. Comparison of four-class classification performance between the full-sample primary analysis and the sensitivity analysis excluding 13 MDD cases with discordant manic symptom ratings. Values are reported as mean ± SD across the 10 outer test folds of nested cross-validation. Recall and F1-score are macro-averaged across the four diagnostic groups, and AUC is calculated using the macro-averaged one-vs-rest strategy. MDD, major depressive disorder; SVM, support vector machine; MLP, multilayer perceptron; AUC, area under the receiver operating characteristic curve; OvR, one-vs-rest.

**Model**	**Analysis**	**Sample size**	**Accuracy**	**Recall (macro)**	**F1 (macro)**	**AUC (OvR)**
SVM	Primary analysis	414	0.60 ± 0.07	0.60 ± 0.05	0.59 ± 0.07	0.86 ± 0.02
SVM	Sensitivity analysis	401	0.58 ± 0.04	0.58 ± 0.04	0.57 ± 0.04	0.86 ± 0.03
Random Forest	Primary analysis	414	0.64 ± 0.06	0.64 ± 0.06	0.63 ± 0.06	0.87 ± 0.02
Random Forest	Sensitivity analysis	401	0.62 ± 0.04	0.62 ± 0.04	0.61 ± 0.05	0.87 ± 0.02
MLP	Primary analysis	414	0.58 ± 0.06	0.58 ± 0.06	0.57 ± 0.06	0.81 ± 0.03
MLP	Sensitivity analysis	401	0.55 ± 0.05	0.55 ± 0.05	0.55 ± 0.04	0.81 ± 0.04
XGBoost	Primary analysis	414	0.59 ± 0.09	0.59 ± 0.09	0.58 ± 0.09	0.83 ± 0.03
XGBoost	Sensitivity analysis	401	0.58 ± 0.07	0.58 ± 0.08	0.57 ± 0.07	0.83 ± 0.03

Section S9 Software Environment

Data preprocessing was conducted using MATLAB (MathWorks, Natick, MA, USA) with the HOMER2 toolbox. Feature extraction, graph metric calculations, and machine learning analyses were implemented in Python. The key software and library versions utilized in this study are listed below:

- **MATLAB**: R2024a
- **HOMER2**: v2.8
- **BrainNet View**: v1.7
- **Python**: 3.10.0
- **Tensorflow**: 2.20.0
- **scikit-learn**: 1.5.2
- **NirSpark**: 1.8.9
